# Age-, sex-, and pathology-related variability in brain structure and cognition

**DOI:** 10.1038/s41398-023-02572-6

**Published:** 2023-08-14

**Authors:** Dario Bachmann, Andreas Buchmann, Sandro Studer, Antje Saake, Katrin Rauen, Isabelle Zuber, Esmeralda Gruber, Roger M. Nitsch, Christoph Hock, Anton Gietl, Valerie Treyer

**Affiliations:** 1 Institute for Regenerative Medicine, University of Zurich, Schlieren, Switzerland; 2 Department of Health Sciences and Technology, ETH Zurich, Zurich, Switzerland; 3 Department of Geriatric Psychiatry, Psychiatric Hospital Zurich, University of Zurich, Zurich, Switzerland; 4 Neuroscience Center Zurich, University of Zurich, Zurich, Switzerland; 5 Neurimmune AG, Schlieren, Switzerland; 6 Department of Nuclear Medicine, University Hospital Zurich, University of Zurich, Zurich, Switzerland

**Keywords:** Molecular neuroscience, Predictive markers, Pathogenesis

## Abstract

This work aimed to investigate potential pathways linking age and imaging measures to early age- and pathology-related changes in cognition. We used [18F]-Flutemetamol (amyloid) and [18F]-Flortaucipir (tau) positron emission tomography (PET), structural MRI, and neuropsychological assessment from 232 elderly individuals aged 50–89 years (46.1% women, 23% APOE-ε4 carrier, 23.3% MCI). Tau-PET was available for a subsample of 93 individuals. Structural equation models were used to evaluate cross-sectional pathways between age, amyloid and tau burden, grey matter thickness and volumes, white matter hyperintensity volume, lateral ventricle volume, and cognition. Our results show that age is associated with worse outcomes in most of the measures examined and had similar negative effects on episodic memory and executive functions. While increased lateral ventricle volume was consistently associated with executive function dysfunction, participants with mild cognitive impairment drove associations between structural measures and episodic memory. Both age and amyloid-PET could be associated with medial temporal lobe tau, depending on whether we used a continuous or a dichotomous amyloid variable. Tau burden in entorhinal cortex was related to worse episodic memory in individuals with increased amyloid burden (Centiloid >12) independently of medial temporal lobe atrophy. Testing models for sex differences revealed that amyloid burden was more strongly associated with regional atrophy in women compared with men. These associations were likely mediated by higher tau burden in women. These results indicate that influences of pathological pathways on cognition and sex-specific vulnerabilities are dissociable already in early stages of neuropathology and cognitive impairment.

## Introduction

Brain pathologies and changes in brain structure are commonly seen in aging [1]. The two main age-related pathological changes are related to cerebrovascular disease and Alzheimer’s disease (AD) [2]. These pathologies often develop simultaneously [3, 4] and explain a substantial proportion of cognitive impairment in older age [2, 5]. Importantly, both neuropathological changes can be identified decades before clinical symptoms occur [6, 7] and emerging evidence suggests that AD- and vascular-related pathologies can have detrimental effects on brain structure and cognition already at low pathology burden [8–11]. Thus, studying healthy individuals to identify and distinguish age- and pathology-related changes in cognition in the earliest stages will pave the way to earlier detection, treatment, and preventive strategies [12, 13].

AD and cerebrovascular pathologies have differential impact on brain health early in the disease process. In AD, amyloid (Aβ) pathology has been shown to trigger tau-mediated neuronal death, thereby altering grey matter structure [14]. The deposition of tau in the medial temporal lobe (MTL), specifically in the entorhinal cortex, is consistently found in cognitively healthy older individuals, including those without concurrent Aβ pathology [15–17]. Tau pathology is closely related to local cortical atrophy [18, 19] and the pattern of tau accumulation and neurodegeneration mirror cognitive domain-specific dementia symptoms in later disease stages [20]. Cerebrovascular pathologies are commonly observed as white matter hyperintensities (WMHs) on MRI scans. The underlying pathology of WMHs mostly reflects demyelination and axonal loss as a consequence of chronic ischaemia [21]. WMHs may be clinically silent in many individuals but increasing WMH volume is associated with cognitive impairment and AD [22]. Although there are exceptions [23], it is commonly observed that AD-related and cerebrovascular-related pathological processes target distinct cognitive domains. Episodic memory (MEM) relies on MTL structures that are preferentially affected in early stages of AD [5, 24]. In turn, decline in executive function (EXE) are often observed together with cerebrovascular pathologies and are associated with frontal-striatal atrophy [5, 24]. Because cognitive decline in different domains generally occurs together as individuals age [25], it is important to examine age- and disease-related brain changes and cognitive decline in the same context to assess the extent to which they are independent of each other [3, 25].

We used multimodal imaging and statistical frameworks for modeling complex relationships among multiple variables [26] to simultaneously quantify early age- and pathology-related changes in cognition. We hypothesized that already low amounts of brain pathologies in individuals who may be considered “normal aging” carry meaningful information when domain- and sex-specific vulnerabilities are considered. Specifically, we demonstrate how Aβ, tau, WMH volume, lateral ventricle volume, and volume/thickness of five a priori-defined brain regions associate with MEM and EXE through multiple pathways and how age acts as a common factor influencing all investigated variables.

## Methods

### Study participants

Study participants are part of the ID-cog cohort, an ongoing prospective cohort study at the University of Zurich, Switzerland that started in 2016. The participants are volunteers recruited through newspaper advertisements. To be enrolled in the study, participants had to be at least 50 years of age and German-speaking. Exclusion criteria included inadequate visual and auditory capacities for neuropsychological assessment, presence of clinically significant depression, presence of a medical condition that is seen as the predominant cause of cognitive impairment (e.g., history of stroke), and presence of diseases that would interfere with study procedures in subsequent years. The study was approved by the ethics committee of the Canton Zurich. All participants gave written informed consent prior to the first study procedure.

We recruited 179 cognitively unimpaired (CU) participants and 54 participants with mild cognitive impairment (MCI). The diagnosis of CU or MCI was made following published diagnostic guidelines [27]. In the present study, we included all participants who had both Aβ-PET imaging, MRI, and APOE genotype assessment, leading to the exclusion of one participant due to missing APOE genotype information. Starting in 2017, a subsample of 93 participants of this cohort obtained tau-PET imaging in addition to Aβ-PET. The median time difference between Aβ-PET and tau-PET imaging was 12 months (range: 0–38 months). For analyses involving both Aβ and tau-PET, we used the T1 image closest to the tau-PET scan (maximum 6 months prior to tau-PET). Baseline neuropsychological data obtained during the Aβ-PET acquisition visit were used in all analyses because an additional neuropsychological examination during the tau-PET acquisition visit was not performed in all participants.

### Demographic and clinical data

Data on age and sex (self-reported as women or men) were ascertained at the clinical visit. APOE genotyping was performed by commercially available Sanger Sequencing (Microsynth AG). Participants were dichotomized into individuals carrying at least one copy of the ε4 allele and APOE4 non-carriers.

### PET/MRI acquisition

We acquired MR and PET images on a 3T Signa PET/MR GE Healthcare scanner. Aβ-PET images were acquired from 90 to 110 min post-injection using approximately 140MBq [18F]-flutemetamol with 4 frames of 5 min each. Tau-PET images were acquired from 80 to 100 min post-injection with 10mCi of [18F]-flortaucipir. A BRAVO 3D T1 MRI sequence (8-channel coil) with voxel size 1 mm in sagittal slice orientation, repetition time (TR) = 8.4 ms, echo time (TE) = 3.2 ms, inversion time (TI) = 450 ms, and flip angle = 12° was acquired in parallel to PET acquisition together with the 3D T2 weighted FLAIR image. A standard 3D CUBE FLAIR sequence with voxel size of 0.48 × 0.48 × 0.6 mm was acquired sagittal with TR/TE/TI = 6502/130.7/1962 ms and flip angle = 90°.

A standard high-resolution T1-weighted fast spoiled gradient recalled acquisition (FSPGR) with inversion recovery scanned on a 750W 3T (32-channel coil) or Premier 3T (48-channel coil) scanner was used for brain parcellation (0.5 mm isotropic voxel size, axial slice orientation, TR/TE/TI = 11/5.2/600 ms, flip angle = 8°).

### T1 and FLAIR MRI processing

Cortical thickness measurements were obtained by processing FSPGR 3D T1-weighed images with FreeSurfer image analysis pipelines (version 7.1.1 for CentOS8 (Linux), surfer.nmr.mgh.harvard.edu). FreeSurfer parcellation of each participant was visually inspected for accuracy, and segmentation errors were manually corrected. Cortical thickness regions of interest (ROIs) included entorhinal cortex, parahippocampal cortex, and mean cortical thickness of a large neocortical composite ROI [28], hereafter referred to as NEOcomp. Additional ROIs were volumes of the hippocampus and striatum (average of putamen and nucleus caudatus). These ROIs were selected because they are likely mediators of age-related variation in cognition and were previously used in a study with a similar research question than the current study [3]. Lateral ventricle volume was included as an additional potential predictor of cognitive performance in the elderly [29, 30]. Thickness and volume measures were averaged across left and right hemisphere estimates. Detailed composition of each ROI by FreeSurfer label is shown in SFig. 1.

White matter lesion volume was estimated from T2-weighted FLAIR images. WMH were segmented on FLAIR images using the lesion prediction algorithm as implemented in the LST toolbox version 3.0.0 (www.statistical-modelling.de/lst.html) for SPM. Lesion masks were created by binarizing lesion probability maps at a threshold of 0.65. An optimal threshold was selected after applying four predefined thresholds (0.3; 0.5; 0.65; 0.75) to 20 randomly selected subjects and visually expecting the generated binarized lesion masks for accuracy. WMH clusters smaller than 2.5 mm^3^ were removed. The minimum cluster is defined as 80% of the smallest lesion size that was consistently detected by three manual raters in a study comparing manual and automated lesion segmentation [31]. Finally, lesion masks were visually inspected and corrected if necessary. In eight participants, WMH volume estimation was not possible due to artifacts on the FLAIR image.

### PET processing

We used PMOD NeuroTool (Version 3.9 and 4.1, PMOD Technologies LLC) for processing and analyzing Aβ-PET and tau-PET images together with the corresponding anatomy from the BRAVO sequence. For Aβ-PET, a global neocortical standardized uptake value ratio (SUVR) was estimated from [18F]-flutemetamol uptake in a large cortical composite ROI, which includes frontal, temporal, and parietal cortices and precuneus. The cerebellar grey matter was used as reference region. For descriptive purposes and certain analyses, we used two previously established Centiloid [32] (CL) cut-off values that mark two relevant inflection points denoting different stages of Aβ pathology [33]: a CL of 12 that marks the transition from the absence of pathology to subtle pathology; and a CL of 30 that marks the presence of established pathology.

The tau-PET scan was coregistered to the participant’s T1-weighted MRI scan using rigid body registration. FreeSurfer parcellation of the T1-weighted MRI scan was then applied to the PET data to extract mean regional [18F]-flortaucipir retention. Average uptake was calculated for a MTL ROI that covered bilateral entorhinal cortex and amygdala and for a neocortical (NEO) ROI that covered bilateral inferior temporal and middle temporal gyri [34]. These regions were selected because most normal elderly adults demonstrate elevated binding confined to the MTL, whereas neocortical binding, particularly in the inferior temporal lobe, is often associated with clinical impairment and the presence of Aβ [34]. An eroded inferior cerebellar grey matter mask was used as the reference region [35]. We report results of analyses using non-partial volume-corrected tau PET measurements but note that the results are virtually identical for partial volume-corrected data (geometric transfer matrix method). Off-target binding was addressed in a sensitivity analysis. For this purpose, we created a skull/meninges mask surrounding the brain (SFig. 6) as previously described [36] to control for a potential effect of off-target binding in the skull/meninges.

### Neuropsychological examination

Participants completed a battery of neuropsychological tasks. We assessed performance in EXE, MEM, visual construction, and working memory using 16 cognitive tasks as previously described [37]. We converted each individual test score to z-scores using the mean and standard deviation of the cohort. Composite scores for each domain were obtained by averaging the corresponding test z-scores.

### Statistical analysis

**Fig. 1:**
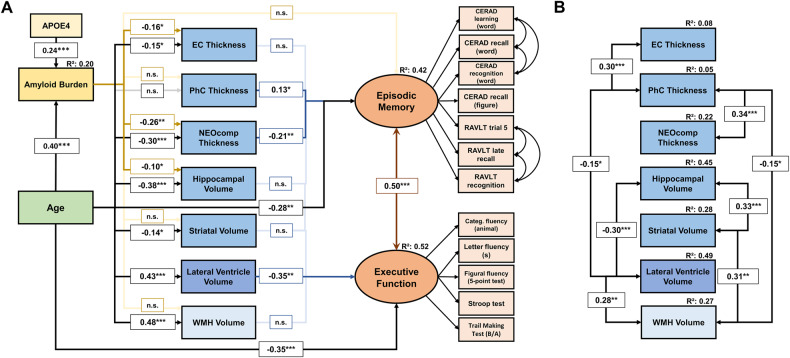
Cognitive domain structural equation model demonstrating a near-universal effect of age on all variables in the model and the mediating roles of pathological and structural measures on cognitive performance in episodic memory and executive function. **A** shows significant and non-significant associations in the specified model. Solid lines represent significant paths at ^*^P < 0.05, ^**^P < 0.01, and ^***^P < 0.001. A continuous amyloid burden variable was included in the presented model. The left-right-headed arrow indicates the residual covariance between episodic memory and executive function performance or correlated residuals of indicators. Indicators were allowed to correlate as the same words were used in these tasks. Residual correlations among indicator variables were highly significant (all *P*’s < 0.001). In B, significant residual covariances among mediating variables are shown. All model parameter estimates are standardized. The R^2^ values denote the variance of the corresponding variables that the model was able to explain. n.s. non-significant, EC entorhinal cortex, PhC parahippocampal cortex. *N* = 232.

We specified a series of models that describe pathways to cognitive function with increasing specificity. Structural equation modeling was used to test the validity of the hypothesized age- and pathology-related pathways on cognitive performance. The following paths were included in models including Aβ but not tau-PET: age was specified as a predictor of global Aβ burden, entorhinal cortex thickness, parahippocampal cortex thickness, NEOcomp thickness, hippocampal volume, striatal volume, lateral ventricle volume, WMH volume, and cognitive performance. Aβ burden was specified as predictor of entorhinal cortex thickness, parahippocampal cortex thickness, NEOcomp thickness, hippocampal volume, and striatal volume. As there is evidence that Aβ pathology may contribute to WMH volume [21], Aβ burden was also specified as a predictor of WMH volume. All structural and pathological measures were specified as predictors of cognitive performance. Cognitive performance was measured using latent variables, similar to our previous work [37]. Here, a latent variable represents the commonality of all neuropsychological tests assigned to it and thus reduces the influence of measurement errors inherent in each individual test [26]. Neuropsychological tests and composite scores assigned to each construct are indicated in Fig. 1A and STable 3.

In a first model, global cognition was set as outcome variable (named *global cognition model*). Then, the global cognition variable was replaced with MEM and EXE latent variables (*cognitive domain model*). We focused on MEM and EXE cognitive domains because we suspected distinguishable pathways towards impairment in the two domains [5, 24]: AD-related pathways towards memory impairment and vascular-related pathways toward executive function impairment. Based on the result of this model, but also considering previous literature [24], we subsequently split the model into an *MEM* and *EXE sub-model* to investigate sex differences. Sex differences were examined in these sub-models after establishing measurement invariance of the MEM and EXE constructs (details in Supplementary Materials). Finally, MTL and NEO tau were added to the MEM sub-model (*tau sub-model*). Given that Aβ-PET signal is no longer associated with atrophy measures after accounting for tau-PET signal [19], Aβ burden was specified as a predictor of MTL and NEO tau, but no longer as a predictor of structural measures. MTL tau was specified as predictor of entorhinal and parahippocampal thickness, and hippocampal volume. Based on evidence suggesting tau in the entorhinal cortex is associated with MEM independently of atrophy [38, 39], MTL tau was also specified as direct predictor of MEM performance. NEO tau was specified as a predictor of NEOcomp thickness but not as a direct predictor of MEM performance.

All models were also assessed using a dichotomized rather than a continuous Aβ load variable using a cut-off of 12 CL. The following covariates were included in all models: effects on cognitive performance were adjusted for years of education, effects of volume measures were adjusted for total intracranial volume, and all effects were adjusted for sex. Time between Aβ and tau-PET was included as a covariate in models including tau-PET measures.

We conducted the following sensitivity analysis: (1) we used the *olsrr* package to identify participants that were both outlier and leverage cases. Unless reported otherwise, results remained unchanged when these influential cases were removed. (2) Models were assessed when tau off-target binding in the skull/meninges mask is included as a covariate.

Analyses were performed in R version 4.0.2. Model assumptions were checked using the *olsrr* package. Models were estimated using robust maximum likelihood estimation in the *lavaan* package. All continuous variables were standardized prior to model entry. Full information maximum likelihood estimation was used to handle missing values. Fit indices for all models can be found in STable 10.

## Results

### Sample characteristics

The characteristics of the total cohort and the sub-cohort with an additional tau-PET scan are summarized in Table 1. Sex-stratified characteristics for the total and tau-PET cohort are provided in STables 1 and 2, respectively.

**Table 1:** Characteristics of all participants with Aβ-PET and sub-group with additional tau-PET.

	Aβ-PET (*n* = 232)	Aβ and tau-PET (*n* = 93)
Age, years mean (SD) [range]	66.4 (8.2) [50-89]	66.4 (7.9) [50-84]
Women, *n* (%)	107 (46.1)	39 (41.9)
Education, years mean (SD)	15.5 (2.9)	16.2 (2.8)
APOE-ε4 carriers, *n* (%)	54 (23.3)	19 (20.4)
History of Hypertension, *n* (%)	73 (31.5)	27 (29.0)
MCI, *n* (%)	54 (23.3)	20 (21.5)
MMSE, mean (SD)	29.1 (1.2)	29.1 (1.1)
Working Memory, mean (SD)	0 (0.79)	0.13 (0.84)
Visual Construction, mean (SD)	0 (0.8)	0.12 (0.74)
MEM, mean (SD)	0.02 (0.8)	0.09 (0.73)
EXE, mean (SD)	0.01 (0.66)	0.06 (0.62)
CL > 12, *n* (%)	77 (33.2)	28 (30.1)
CL > 30, *n* (%)	27 (11.6)	8 (8.6)

Standardized composite scores for cognitive domains in the tau sub-cohort are based on the full cohort. *SD* standard deviation, *MCI* mild cognitive impairment, *MMSE* Mini-Mental State Examination, *MEM* episodic memory, *EXE* executive function, *CL* Centiloid.

### Associations between age, structural, and pathological measures

We first report the effects of age and Aβ pathology on structural and pathological measures. These estimates are practically identical for the global cognition model and the cognitive domain model. Significant estimates for these associations are shown in Fig. 1A. Significant residual covariances among mediating measures are shown in Fig. 1B. Complete model coefficients are provided in STable 3. The model demonstrates that age is a significant predictor of almost all structural and pathological measures. Only the association between age and parahippocampal cortex thickness did not reach our significance threshold (β = −0.13; 95% CI, −0.26 to 0.003; *P* = 0.06). Estimating the model with CU participants only showed that Aβ pathology was no longer associated with entorhinal cortex thickness (*P* = 0.72) and hippocampal volume (*P* = 0.07). Higher Aβ burden was still associated with lower global NEOcomp thickness. When we replaced the continuous Aβ variable with a dichotomous Aβ variable, we observed similar results as in the model with CU participants only, suggesting that once Aβ can be linked to MTL atrophy, individuals are more likely to be in the MCI stage.

### Effects on global cognition, episodic memory and executive function

Lateral ventricle volume (β = −0.34; 95% CI, −0.55 to −0.13; *P* = 0.002) and age (β = −0.27; 95% CI, −0.43 to −0.10; *P* = 0.002) were the only measures that were significantly associated with global cognition. Both associations remained comparable when the model was estimated with CU participants only (STable 4). Age was associated with worse performance in MEM and EXE domains (Fig. 1A). After accounting for the effects of the predictor variables, the cognitive domains still showed strong and highly significant residual covariance. Of the structural and pathological measures that were not associated with cognitive measures, paths from Aβ burden (*P* = 0.07) and hippocampal volume (*P* = 0.10) to MEM and striatal volume (*P* = 0.10) to EXE were closest to the significance threshold. When the model was estimated with CU participants only, parahippocampal cortex thickness was no longer associated with MEM whereas increased lateral ventricle volume was still associated with poorer EXE. WMH volume was not associated with cognitive performance in any of the models. It is worth noting that despite its higher complexity, the cognitive domain model provided a considerably better fit to the data than the global cognition model (STable 10).

### Sex differences in episodic memory and executive function sub-model

**Fig. 2:**
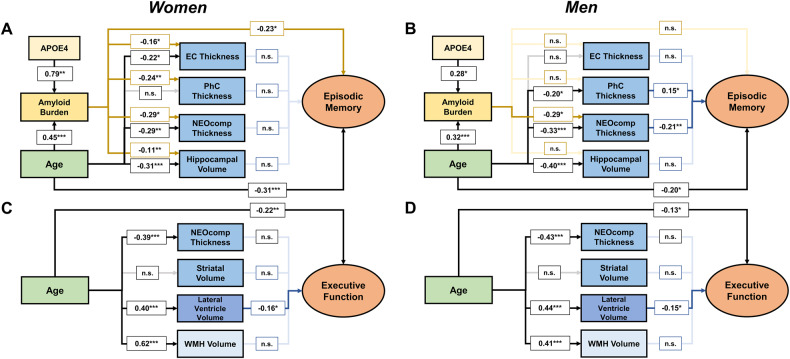
Multigroup analyses suggest sex differences in the MEM sub-model but not in the EXE sub-model. Results are shown on separate models for women (*N* = 107) in **A** and **C** and for men (*N* = 125) in **B** and **D**. Model parameter estimates are not standardized, so that the estimates for the same path in the MEM sub-model and EXE sub-model, respectively, can be compared between men and women. A continuous Aβ burden variables was included in the presented model. For a simplified representation, indicators of the latent constructs were removed. n.s. non-significant, EC entorhinal cortex, PhC parahippocampal cortex. ^*^P < 0.05, ^**^P < 0.01, ^***^P < 0.001.

To investigate sex-differences, we split the cognitive domain model into MEM and EXE sub-models. Figure 2 shows the results for women (A and C) and men (B and D) in separate models. Complete model coefficients and correlation plots are provided in STables 6 and 7 and SFig. 2, respectively. Overall, these models demonstrate that in the MEM sub-model, increased Aβ burden is more closely related to atrophy measures and MEM in women, whereas in men, atrophy measures are more closely related to MEM. However, constraining single paths to be equal for both sexes did not significantly decrease the model fit to data (data not shown), implying that the model fits equally well when paths for men and women are estimated as one. We identified 9 influential cases (4 in EXE sub-model, 5 in MEM sub-model) whose removal led to changes in some parameter estimates. With one exception (61 years), influential cases were older than 74 years. In women, the association between Aβ burden and entorhinal cortex thickness was no longer significant after excluding influential cases (*P* = 0.079). In men, some associations between structural measures and cognition became stronger. Most notable, in addition to parahippocampal cortex thickness also hippocampal volume was associated with MEM (*P* = 0.028).

### Associations between Aβ-PET, Tau-PET, and cognition in Tau sub-model

**Fig. 3:**
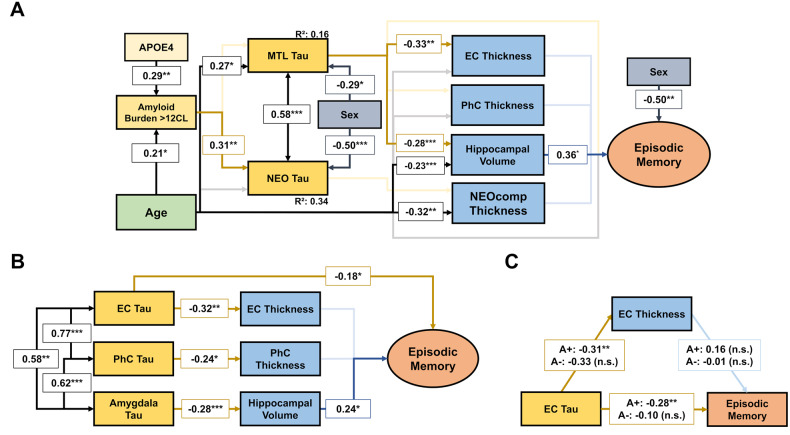
Direct and mediating relationships among age, Aβ burden, tau burden, thickness/volume of brain measures, and episodic memory. **A** depicts the tau sub-model and shows the differential association between age, Aβ, and tau when a dichotomous amyloid variable is used. Female sex was associated with better episodic memory performance and increased tau pathology particularly in the NEO Tau composite ROI. A significant association between sex and Aβ burden was found as well (β = 0.28, *P* = 0.001), but this was not included in the model for ease of visualization. “Sex” was coded as women = 0 and men = 1. In **B**, MTL regions were examined separately in one model. Tau load in the amygdala, not the hippocampus, was used as a predictor of hippocampal volume because the amygdala is less affected by [18F]-flortaucipir off-target binding in the choroid plexus. The moderated mediation model in **C** indicates the association between EC tau, EC thickness and an episodic memory composite score in Aβ positive (A+; CL > 12) and Aβ negative (A-; CL < 12) participants. Despite being of similar magnitude, the association between EC tau and EC thickness did not reach significance in Aβ negative participants (*P* = 0.09). Parameter estimates are unstandardized in B and C so that estimates on the same path can be compared. n.s. non-significant, EC entorhinal cortex, PhC parahippocampal cortex. ^*^P < 0.05, ^**^P < 0.01, ^***^P < 0.001. *N* = 93.

Figure 3A shows the results of the tau sub-model when a dichotomous Aβ variable is used. Complete model coefficients and correlation plots are provided in STables 8 and 9 and SFigures 3, 4, and 5, respectively. The model demonstrates that elevated Aβ burden is associated with increased NEO tau but not with MTL tau whereas higher age is associated with increased MTL tau but not with NEO tau. When we used a continuous Aβ variable we found that Aβ, but not age, is associated with increased MTL tau (Table 2). However, these associations were dependent on 3 influential cases with high Aβ burden (CL > 71), so that when these cases were excluded, neither Aβ nor age was associated with MTL tau. Associations of age and Aβ with NEO tau did not change when influential cases were excluded. Sex had strong and highly significant effects on tau burden, such that female sex was associated with increased tau burden in both MTL and NEO ROI, despite that there were fewer women with increased Aβ than men (6/39 women vs. 22/54 men with CL > 12). Using two-sample t-tests, we found significantly higher tau burden in women in both ROIs, also when the analysis was restricted to men and women with CL < 12 (MTL tau: *P* = 0.009; NEO tau: *P* < 0.001). Previous studies suggest that tau in the entorhinal cortex is associated with MEM performance independently of atrophy [38]. To test this possibility, we built a model that specifically examines MTL structures (Fig. 3B). The model demonstrates that entorhinal cortex tau is directly associated with lower MEM performance independent of MTL atrophy. Controlling for age, sex, education, time between PET scans, and hippocampal volume, we found that this association was only observable in individuals with CL > 12 (Fig. 3C). The same path did not reach significance when parahippocampal measures were used in the model (*P* = 0.06).

**Table 2:** Amyloid-to-tau and age-to-tau paths when continuous or dichotomous Aβ variable is used in the structural equation model.

	Continuous amyloid		CL > 12	
Path	β (95% CI)	*P*	β (95% CI)	*P*
Amyloid to MTL Tau	0.494 (0.172 to 0.816)	0.003	0.086 (−0.510 to 0.682)	0.778
Amyloid to NEO Tau	0.464 (0.108 to 0.819)	0.011	0.675 (0.215 to 1.134)	0.004
Age to MTL Tau	0.056 (−0.153 to 0.265)	0.598	0.266 (0.061 to 0.472)	0.011
Age to NEO Tau	0.084 (−0.107 to 0.274)	0.389	0.222 (−0.024 to 0.468)	0.077

Model parameter estimates for both models are unstandardized so that estimates for the same path can be compared across models.

### Tau-PET off-target binding

[18F]-flortaucipir off-target binding in the meninges/skull has been found previously particularly in women [36, 40]. Similarly, we found higher off-target binding in meninges/skull in women compared with men (SFig. 7). Including [18F]-flortaucipir uptake in the skull mask as a covariate in the tau sub-model did not change the results reported above.

## Discussion

Our structural equation models show that in middle-aged to older adults, age is the main contributor to cross-sectional structural, pathological, and cognitive variability whereas structural or pathological measures themselves show subtle or even absent associations with cognition. Although MEM and EXE performance were highly correlated with each other, AD-related pathways involving Aβ and tau pathology, and medial temporal structures selectively affected MEM performance. In contrast, EXE was exclusively related to lateral ventricle volume, a marker that is dominated by widespread tissue loss throughout the brain and itself strongly correlated with age. Furthermore, we identified sex as an important modifier of many associations. Finally, our results suggest that the effect of age on MTL tau may be twofold: a modest age-related increase in MTL tau detectable when Aβ is low, and a pronounced AD-related increase mediated by increased Aβ pathology.

A central aspect of this work was to investigate the contribution of age to pathologies, brain atrophy, and cognition. Age was a significant and consistent contributor to almost all investigated structural and pathological measures and showed similar associations with MEM and EXE performance. Associations of age with WMH and lateral ventricle volume were particularly strong. Age showed also strong associations with cortical thickness in a large neocortical ROI (NEOcomp ROI) and hippocampal volume but this was not the case for entorhinal or parahippocampal cortex thickness, similar to previous cross-sectional analyses [41–43]. Longitudinally, we would expect accelerated atrophy of MTL structures with advancing age [13, 42, 43]. Additionally, age was associated with structural measures through the mediating influence of Aβ pathology. MCI participants drove the associations between Aβ and MTL structure volume/thickness, but not the association with NEOcomp thickness. Fjell and colleagues have previously pointed out Aβ-atrophy relationships that differ in CU and MCI [13]. The authors speculated that Aβ may reflect processes related to brain changes that can be compensated for in the cortex. Once these processes reach the MTL, the likelihood of an MCI diagnosis increases because compensation in this region is more difficult [13].

Tau pathology mediates the relationship between Aβ and atrophy measures [14, 19]. This is reflected in the tau sub-model in which MTL tau was strongly associated with entorhinal cortex thickness and hippocampus volume. Although continuous Aβ was strongly associated with MTL tau, our sensitivity analysis indicates that the association was driven by three influential cases, all of which had CL > 71. This is consistent with our finding that age and Aβ burden were inconsistently associated MTL tau burden. If a continuous Aβ variable was used, Aβ but not age was associated with MTL tau, whereas if a dichotomous Aβ variable was used, age but not Aβ was associated with MTL tau. Autopsy studies previously showed that MTL tau pathology is related not only to Aβ pathology but also to age [44], and revealed that MTL neurofibrillary tangles in the elderly are essentially universal even in the absence of Aβ pathology [45]. Imaging studies also suggest that tau in the entorhinal cortex, and possible outside MTL regions, may accumulate independently of Aβ and gradually increases with age [39, 46–48]. This association can be observed in individuals with low Aβ pathology but less so in individuals with increased Aβ pathology [46], as higher Aβ itself is associated with both exacerbated tau accumulation in the MTL and advanced age.

In our models, Aβ pathology showed a robust association with tau in NEO ROI, consistent with previous studies that found the strongest AD-related tau accumulation in inferior temporal lobe [49]. The presence of Aβ and tau in this region is associated widespread neocortical tau accumulation and increased risk for progression to MCI [34, 50]. Previous work found that Aβ in the CL range of 15-18 robustly predicts future cognitive decline [11]. Similarly, we found that in individuals with CL > 12, tau pathology in the entorhinal cortex is linked to subtle MEM alterations independent of MTL atrophy. This is also consistent with studies indicating that tau-PET is a better predictor of longitudinal cognitive decline than MRI-based cortical thickness in CU individuals [51] and that tau pathology affects memory performance in early disease stages predominantly via aberrant MTL functional connectivity [38]. It is conceivable that a direct relationship between MTL tau and memory loss may be observed in the absence of Aβ, although evidence regarding such an effect is inconclusive [52, 53]. Overall, our results support a model in which tau is in part an aging phenomenon [44, 45, 48], characterized by gradual accumulation in the absence of Aβ, particularly in MTL regions [46]. This may be related to primary age-related tauopathy (PART) [54], which by itself is also related to MTL atrophy and memory decline, albeit to a lesser extent than in the presence of Aβ [52, 54, 55]. In individuals with elevated Aβ pathology, tau pathology in the entorhinal cortex is associated with subtle MEM alterations independently of MTL atrophy [38, 53, 56] and later with the spread of tau into the temporal neocortex and MTL atrophy [46]. If MTL atrophy is associated with memory performance, a diagnosis of MCI is likely [13, 38].

In addition to age, another focus was on potential sex differences, as susceptibility to AD and cerebrovascular pathological processes may differ between men and women [57]. We speculated that connections among variables in the EXE sub-model are stronger in males whereas females might show stronger connections in the MEM sub-model. While no prominent sex differences in the EXE sub-model were observed, we found that sex moderates the associations between Aβ burden and cortical structures and MEM. The tau sub-model suggests that the increased tau load observed in women mediates the effect of Aβ on atrophy. While this interpretation would be in line with previous studies [58], we did not examine an interactive effect between sex and Aβ pathology on tau burden because Aβ pathology in females in the tau sub-cohort was generally low (only 6 with CL > 12). A cohort of similar age to ours showed no such interaction [59], and we found increased tau burden in women even if we examined sex differences only in those participants with CL < 12. Thus, we would not rule out the possibility that there are Aβ-independent drivers of tau pathology in late middle-aged females as has been suggested previously [60]. Interestingly, despite the higher tau burden in women, sex differences in thickness or volume of brain regions were minimal or absent but linking these structures with Aβ burden is nevertheless possible in women. One explanation could be that factors not necessarily related to AD contribute more strongly to brain atrophy in men as they age, which may have led to greater variability in cortical thickness in men. For instance, increased vascular risk was associated with lower cognitive performance in men but not in women in our previous analysis, which was based on the same cohort [37]. This association could be mediated by brain atrophy, which was associated with MEM in men but not in women in the present analysis. Other imaging studies also suggest that compared to men, women are more resistant to the detrimental effects of tau pathology as they measured higher cross-sectional cortical thickness at similar tau burden [61]. Finally, we found that women showed significantly better MEM performance. This observation is not unexpected, especially since our MEM variable focuses on verbal memory performance [62], but it underscores the need for sex-specific standards for verbal memory testing, as the memory advantage seen in women can mask early signs of AD and delay the diagnosis of MCI [63].

Our results need to be interpreted in light of the study cohort. Participants in this study are volunteers who were recruited via newspaper advertisements and were selected to represent a healthy aging population. Thus, most participants are at an early stage of developing brain pathologies, and many will age without developing dementia. In addition, it is reasonable to assume that participants with MCI are in the early stages of the condition. Other studies with similar research questions were able to link age-related cognitive changes much more consistently to underlying brain alterations [3, 64]. However, individuals in these cohorts were older and probably had a greater pathological burden and greater variability in cognitive performance than the present cohort. Our recruitment strategy, coupled with the cross-sectional study design, may also explain the unexpected direction of the association between NEOcomp thickness and MEM. Higher NEOcomp thickness might be significantly associated with poorer MEM performance because older participants in the cohort were increasingly selected to have preserved, well-functioning MEM, but age- and AD-related processes continued to adversely affect NEOcomp thickness. Particularly older individuals with high Aβ pathology or other pathologies associated with reduced cortical thickness will need to have well-preserved MEM to participate in the study. This in turn would suggest compensatory or reserve mechanisms that allow these individuals to maintain cognitive performance [65].

There are several limitations of this study. The sample size of the sub-group with both Aβ and tau-PET was relatively small. The good model fit to data and the fact that paths in our structural equation models were based on previous literature increase our confidence in the correctness of our results despite the limited sample size. Furthermore, many of our conclusions are consistent with a recent study with a different statistical approach and a novel tau PET-tracer [66]. Nevertheless, we were limited in investigating potentially moderating effects of sex and APOE genotype or Aβ-independent effects of the APOE genotype [46, 67]. Furthermore, as the participants were selected to represent a relatively healthy aging cohort, the variability in Aβ and tau pathology was low with many participants, particularly women in the tau sub-cohort, having very low Aβ pathology. As for our statistical approach, we chose structural equation modeling because it allowed us to examine multiple variables simultaneously; however, this likely weakened the predictive power of many mediating variables in the model due to suppression of their non-shared variance. Furthermore, the individual structures do not act independently of each other to predict cognition. For example, the MTL is a complex system in which the ensemble likely behaves in ways not predicted by its components [68]. Future research should therefore investigate functional properties of these regions and how they respond to structural atrophy and pathological measures [38]. Finally, we took a closer look into AD-related processes as the a priori knowledge of the pathophysiological processes is broader than is the case, for example, with WMH. However, detrimental effects of WMH on brain structure or cognition would likely be detectable even in healthy adults if more regional WMH were studied [69].

In conclusion, we showed that with increasing age, multiple, often sex-modified pathological pathways begin to develop that ultimately may lead to cognitive decline. The contribution of these underlying pathological processes to cognitive alterations were subtle in this cohort and a large proportion of the effect of age on cognition was not mediated by any of the pathological and structural measures included in this analysis. Recognizing these early signs of pathology-related cognitive decline may be critical for selecting individuals for therapies before degenerative processes have progressed too far.

### Supplementary information

Supplementary Information

## References

[1] Fjell AM, Walhovd KB (2010). Structural Brain Changes in Aging: Courses, Causes and Cognitive Consequences. Rev Neurosci.

[2] Kapasi A, DeCarli C, Schneider JA (2017). Impact of multiple pathologies on the threshold for clinically overt dementia. Acta Neuropathol.

[3] Hedden T, Schultz AP, Rieckmann A, Mormino EC, Johnson KA, Sperling RA (2016). Multiple Brain Markers are Linked to Age-Related Variation in Cognition. Cereb Cortex.

[4] Vemuri P, Lesnick TG, Knopman DS, Przybelski SA, Reid RI, Mielke MM (2019). Amyloid, Vascular, and Resilience Pathways Associated with Cognitive Aging. Ann Neurol.

[5] Jagust W (2013). Vulnerable neural systems and the borderland of brain aging and neurodegeneration. Neuron.

[6] Vermunt L, Sikkes SAM, van den Hout A, Handels R, Bos I, van der Flier WM (2019). Duration of preclinical, prodromal, and dementia stages of Alzheimer’s disease in relation to age, sex, and APOE genotype. Alzheimer’s Dement.

[7] d’Arbeloff T, Elliott ML, Knodt AR, Melzer TR, Keenan R, Ireland D et al. White matter hyperintensities are common in midlife and already associated with cognitive decline. Brain Commun. 2019; 1. 10.1093/braincomms/fcz041 PMC6928390 31894208

[8] Leal SL, Lockhart SN, Maass A, Bell RK, Jagust WJ (2018). Subthreshold Amyloid Predicts Tau Deposition in Aging. J Neurosci.

[9] Landau SM, Horng A, Jagust WJ (2018). Memory decline accompanies subthreshold amyloid accumulation. Neurology.

[10] Yau W-YW, Shirzadi Z, Yang H-S, Ikoba AP, Rabin JS, Properzi MJ (2022). Tau Mediates Synergistic Influence of Vascular Risk and Aβ on Cognitive Decline. Ann Neurol.

[11] Farrell ME, Jiang S, Schultz AP, Properzi MJ, Price JC, Becker JA (2021). Defining the Lowest Threshold for Amyloid-PET to Predict Future Cognitive Decline and Amyloid Accumulation. Neurology.

[12] Bischof GN, Jacobs HIL (2019). Subthreshold amyloid and its biological and clinical meaning: Long way ahead. Neurology.

[13] Fjell AM, McEvoy L, Holland D, Dale AM, Walhovd KB (2014). What is normal in normal aging? Effects of aging, amyloid and Alzheimer’s disease on the cerebral cortex and the hippocampus. Prog Neurobiol.

[14] Long JM, Holtzman DM (2019). Alzheimer Disease: An Update on Pathobiology and Treatment Strategies. Cell.

[15] Braak H, Braak E (1997). Frequency of stages of Alzheimer-related lesions in different age categories. Neurobiol aging.

[16] Maass A, Landau S, Baker SL, Horng A, Lockhart SN, La Joie R (2017). Comparison of multiple tau-PET measures as biomarkers in aging and Alzheimer’s disease. NeuroImage.

[17] Schöll M, Lockhart Samuel N, Schonhaut Daniel R, O’Neil James P, Janabi M, Ossenkoppele R (2016). PET Imaging of Tau Deposition in the Aging Human Brain. Neuron.

[18] Schäfer A, Chaggar P, Thompson TB, Goriely A, Kuhl E (2021). Predicting brain atrophy from tau pathology: a summary of clinical findings and their translation into personalized models. Brain Multiphys.

[19] La Joie R, Visani AV, Baker SL, Brown JA, Bourakova V, Cha J (2020). Prospective longitudinal atrophy in Alzheimer’s disease correlates with the intensity and topography of baseline tau-PET. Sci Transl Med.

[20] Graff-Radford J, Yong KX, Apostolova LG, Bouwman FH, Carrillo M, Dickerson BC (2021). New insights into atypical Alzheimer’s disease in the era of biomarkers. Lancet Neurol.

[21] Prins ND, Scheltens P (2015). White matter hyperintensities, cognitive impairment and dementia: an update. Nat Rev Neurol.

[22] Debette S, Schilling S, Duperron MG, Larsson SC, Markus HS (2019). Clinical Significance of Magnetic Resonance Imaging Markers of Vascular Brain Injury: A Systematic Review and Meta-analysis. JAMA Neurol.

[23] Ossenkoppele R, Pijnenburg YAL, Perry DC, Cohn-Sheehy BI, Scheltens NME, Vogel JW (2015). The behavioural/dysexecutive variant of Alzheimer’s disease: clinical, neuroimaging and pathological features. Brain.

[24] Buckner RL (2004). Memory and Executive Function in Aging and AD: Multiple Factors that Cause Decline and Reserve Factors that Compensate. Neuron.

[25] Salthouse TA (2010). Selective review of cognitive aging. J Int Neuropsychol Soc.

[26] Beran TN, Violato C (2010). Structural equation modeling in medical research: a primer. BMC Res Notes.

[27] Winblad B, Palmer K, Kivipelto M, Jelic V, Fratiglioni L, Wahlund LO (2004). Mild cognitive impairment-beyond controversies, towards a consensus: report of the International Working Group on Mild Cognitive Impairment. J Intern Med.

[28] Fjell AM, McEvoy L, Holland D, Dale AM, Walhovd KB (2013). Initiative AsDN. Brain changes in older adults at very low risk for Alzheimer’s disease. J Neurosci.

[29] Nestor SM, Rupsingh R, Borrie M, Smith M, Accomazzi V, Wells JL (2008). Ventricular enlargement as a possible measure of Alzheimer’s disease progression validated using the Alzheimer’s disease neuroimaging initiative database. Brain.

[30] Jack CR, Shiung MM, Gunter JL, O’Brien PC, Weigand SD, Knopman DS (2004). Comparison of different MRI brain atrophy rate measures with clinical disease progression in AD. Neurology.

[31] Egger C, Opfer R, Wang C, Kepp T, Sormani MP, Spies L (2017). MRI FLAIR lesion segmentation in multiple sclerosis: Does automated segmentation hold up with manual annotation?. Neuroimage Clin.

[32] Klunk WE, Koeppe RA, Price JC, Benzinger TL, Devous MD, Jagust WJ (2015). The Centiloid Project: standardizing quantitative amyloid plaque estimation by PET. Alzheimers Dement.

[33] Salvadó G, Molinuevo JL, Brugulat-Serrat A, Falcon C, Grau-Rivera O, Suárez-Calvet M (2019). Centiloid cut-off values for optimal agreement between PET and CSF core AD biomarkers. Alzheimers Res Ther.

[34] Ossenkoppele R, Pichet Binette A, Groot C, Smith R, Strandberg O, Palmqvist S (2022). Amyloid and tau PET-positive cognitively unimpaired individuals are at high risk for future cognitive decline. Nat Med.

[35] Baker SL, Maass A, Jagust WJ (2017). Considerations and code for partial volume correcting [(18)F]-AV-1451 tau PET data. Data Brief.

[36] Smith R, Strandberg O, Leuzy A, Betthauser TJ, Johnson SC, Pereira JB (2021). Sex differences in off-target binding using tau positron emission tomography. Neuroimage Clin.

[37] Bachmann D, Roman ZJ, Buchmann A, Zuber I, Studer S, Saake A (2022). Lifestyle Affects Amyloid Burden and Cognition Differently in Men and Women. Ann Neurol.

[38] Berron D, Vogel JW, Insel PS, Pereira JB, Xie L, Wisse LEM (2021). Early stages of tau pathology and its associations with functional connectivity, atrophy and memory. Brain.

[39] Maass A, Lockhart SN, Harrison TM, Bell RK, Mellinger T, Swinnerton K (2018). Entorhinal Tau Pathology, Episodic Memory Decline, and Neurodegeneration in Aging. J Neurosci.

[40] Flores S, Chen CD, Su Y, Dincer A, Keefe SJ, McKay NS (2022). Investigating Tau and Amyloid Tracer Skull Binding in Studies of Alzheimer disease. J Nucl Med.

[41] Frangou S, Modabbernia A, Williams SCR, Papachristou E, Doucet GE, Agartz I (2022). Cortical thickness across the lifespan: Data from 17,075 healthy individuals aged 3-90 years. Hum Brain Mapp.

[42] Fjell AM, Westlye LT, Grydeland H, Amlien I, Espeseth T, Reinvang I (2014). Accelerating cortical thinning: unique to dementia or universal in aging?. Cereb Cortex.

[43] Wisse LE, Xie L, Das SR, de Flores R, Hansson O, Habes M (2022). Tau pathology mediates age effects on medial temporal lobe structure. Neurobiol Aging.

[44] Mungas D, Tractenberg R, Schneider JA, Crane PK, Bennett DA (2014). A 2-process model for neuropathology of Alzheimer’s disease. Neurobiol Aging.

[45] Braak H, Thal DR, Ghebremedhin E, Del Tredici K (2011). Stages of the Pathologic Process in Alzheimer Disease: Age Categories From 1 to 100 Years. J Neuropathol Exp Neurol.

[46] Sanchez JS, Becker JA, Jacobs HIL, Hanseeuw BJ, Jiang S, Schultz AP (2021). The cortical origin and initial spread of medial temporal tauopathy in Alzheimer’s disease assessed with positron emission tomography. Sci Transl Med.

[47] Cho H, Lee HS, Choi JY, Lee JH, Ryu YH, Lee MS (2018). Predicted sequence of cortical tau and amyloid-β deposition in Alzheimer disease spectrum. Neurobiol Aging.

[48] Lowe VJ, Wiste HJ, Senjem ML, Weigand SD, Therneau TM, Boeve BF (2018). Widespread brain tau and its association with ageing, Braak stage and Alzheimer’s dementia. Brain.

[49] Insel PS, Young CB, Aisen PS, Johnson KA, Sperling RA, Mormino EC (2022). Tau positron emission tomography in preclinical Alzheimer’s disease. Brain.

[50] Lee WJ, Brown JA, Kim HR, La Joie R, Cho H, Lyoo CH (2022). Regional Aβ-tau interactions promote onset and acceleration of Alzheimer’s disease tau spreading. Neuron.

[51] Ossenkoppele R, Smith R, Mattsson-Carlgren N, Groot C, Leuzy A, Strandberg O (2021). Accuracy of Tau Positron Emission Tomography as a Prognostic Marker in Preclinical and Prodromal Alzheimer Disease: A Head-to-Head Comparison Against Amyloid Positron Emission Tomography and Magnetic Resonance Imaging. JAMA Neurol.

[52] Chen X, Cassady KE, Adams JN, Harrison TM, Baker SL, Jagust WJ (2021). Regional Tau Effects on Prospective Cognitive Change in Cognitively Normal Older Adults. J Neurosci.

[53] Sperling RA, Mormino EC, Schultz AP, Betensky RA, Papp KV, Amariglio RE (2019). The impact of amyloid-beta and tau on prospective cognitive decline in older individuals. Ann Neurol.

[54] Crary JF, Trojanowski JQ, Schneider JA, Abisambra JF, Abner EL, Alafuzoff I (2014). Primary age-related tauopathy (PART): a common pathology associated with human aging. Acta Neuropathol.

[55] Teylan M, Mock C, Gauthreaux K, Chen YC, Chan KCG, Hassenstab J (2020). Cognitive trajectory in mild cognitive impairment due to primary age-related tauopathy. Brain.

[56] Das SR, Xie L, Wisse LEM, Vergnet N, Ittyerah R, Cui S (2019). In vivo measures of tau burden are associated with atrophy in early Braak stage medial temporal lobe regions in amyloid-negative individuals. Alzheimers Dement.

[57] Podcasy JL, Epperson CN (2016). Considering sex and gender in Alzheimer disease and other dementias. Dialogues Clin Neurosci.

[58] Buckley RF, Scott MR, Jacobs HIL, Schultz AP, Properzi MJ, Amariglio RE (2020). Sex Mediates Relationships Between Regional Tau Pathology and Cognitive Decline. Ann Neurol.

[59] Palta P, Rippon B, Tahmi M, Pardo M, Johnson A, Tomljanovic Z (2021). Sex differences in in vivo tau neuropathology in a multiethnic sample of late middle-aged adults. Neurobiol Aging.

[60] Buckley RF, O’Donnell A, McGrath ER, Jacobs HIL, Lois C, Satizabal CL (2022). Menopause status moderates sex differences in tau burden: a Framingham PET Study. Ann Neurol.

[61] Ossenkoppele R, Lyoo CH, Jester-Broms J, Sudre CH, Cho H, Ryu YH (2020). Assessment of Demographic, Genetic, and Imaging Variables Associated With Brain Resilience and Cognitive Resilience to Pathological Tau in Patients With Alzheimer Disease. JAMA Neurol.

[62] Sundermann EE, Biegon A, Rubin LH, Lipton RB, Mowrey W, Landau S (2016). Better verbal memory in women than men in MCI despite similar levels of hippocampal atrophy. Neurology.

[63] Sundermann EE, Maki P, Biegon A, Lipton RB, Mielke MM, Machulda M (2019). Sex-specific norms for verbal memory tests may improve diagnostic accuracy of amnestic MCI. Neurology.

[64] Yu L, Boyle PA, Leurgans S, Schneider JA, Bennett DA (2014). Disentangling the effects of age and APOE on neuropathology and late life cognitive decline. Neurobiol Aging.

[65] Stern Y, Arenaza-Urquijo EM, Bartrés-Faz D, Belleville S, Cantilon M, Chetelat G (2018). Whitepaper: Defining and investigating cognitive reserve, brain reserve, and brain maintenance. Alzheimer’s Dementia.

[66] Therriault J, Pascoal TA, Lussier FZ, Tissot C, Chamoun M, Bezgin G (2022). Biomarker modeling of Alzheimer’s disease using PET-based Braak staging. Nat Aging.

[67] Young CB, Johns E, Kennedy G, Belloy ME, Insel PS, Greicius MD (2023). APOE effects on regional tau in preclinical Alzheimer’s disease. Mol Neurodegeneration.

[68] Ranganath C, Ritchey M (2012). Two cortical systems for memory-guided behaviour. Nat Rev Neurosci.

[69] Ter Telgte A, van Leijsen EMC, Wiegertjes K, Klijn CJM, Tuladhar AM, de Leeuw FE (2018). Cerebral small vessel disease: from a focal to a global perspective. Nat Rev Neurol.

