# Supplementary material for: Age-, sex-, and pathology-related variability in brain structure and cognition
Source: Transl Psychiatry. 2023 Aug 14;13:278. doi: 10.1038/s41398-023-02572-6 (PMC10423720; doi:10.1038/s41398-023-02572-6)
Supplement: Supplementary file 1 — Supplementary Information [file 41398_2023_2572_MOESM1_ESM.doc]

**Age-, Sex-, and Pathology-Related Variability in Brain Structure and Cognition**

**- Supplementary Materials -**

**
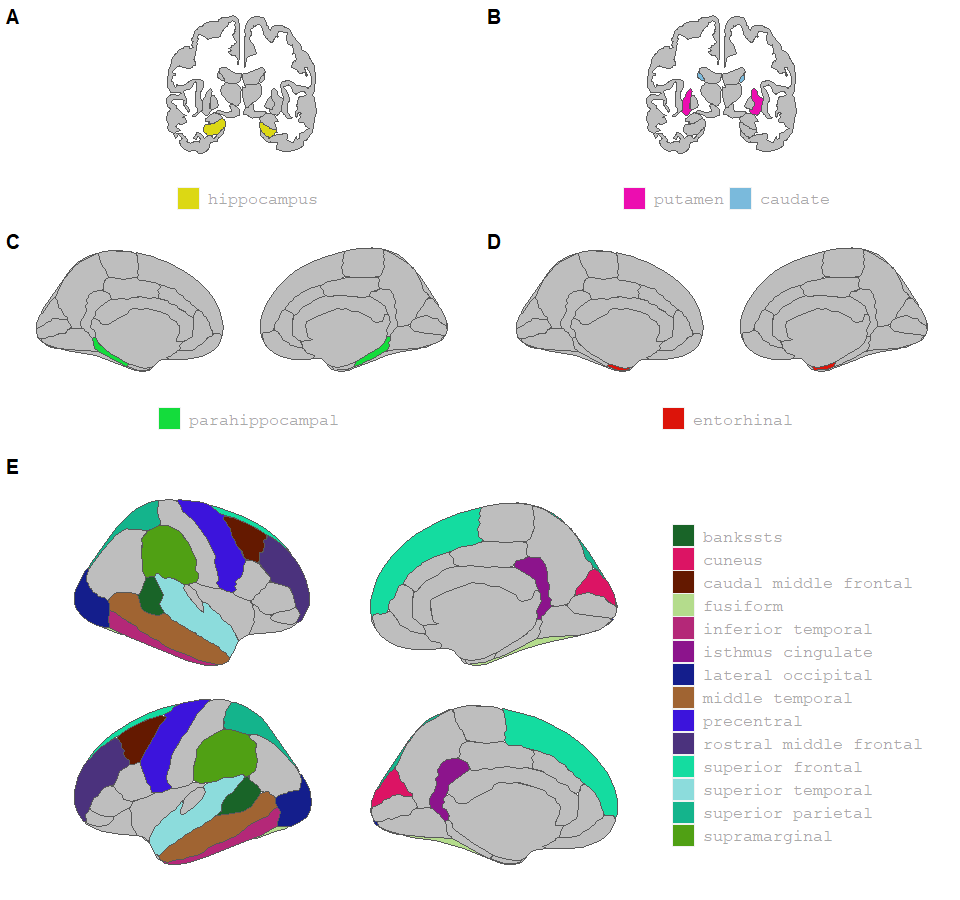
SFigure 1. FreeSurfer regions of interest from the Desikan-Killiany Atlas included in the present analysis.** Regions were chosen because they are likely mediators of age-related differences in cognition and have been previously used by Hedden and colleagues who addressed a similar research question as we did (1).

**STable 1. Study cohort with amyloid-PET scan: description overall and stratified by sex.**

|  | **ALL (n=232)** | **Men (n=125)** | **Women (n=107)** | **Group differences** |
| --- | --- | --- | --- | --- |
| **Age, years mean (SD) [range]** | 66.4 (8.2) [50-89] | 67.7 (7.6) [50-81] | 65.0 (8.8) [50-89] | t=-2.52, df=230, p=0.01 |
| **Education, years mean (SD)** | 15.5 (2.9) | 16.5 (2.6) | 14.3 (2.7) | W=3813, p<0.001 |
| **APOE-ε4 carriers, n (%)** | 54 (23.3) | 24 (19.2) | 30 (28.0) | χ^2^=2.05, df=1, p=0.15 |
| **MCI, n (%)** | 54 (23.3) | 35 (28.0) | 19 (17.8) | χ^2^=2.84, df=1, p=0.09 |
| **MMSE, mean (SD)** | 29.1 (1.2) | 29.1 (1.1) | 29.2 (1.3) | t=1.05, df=230, p=0.29 |
| **BMI, mean (SD)** | 25.4 (3.9) | 24.2 (4.1) | 26.5 (3.3) | W=4136.5, p<0.001 |
| **Diabetes mellitus, n (%)** | 9 (3.9) | 7 (5.6) | 2 (1.8) | χ^2^ =1.27, df=1, p=0.26 |
| **CVD, n (%) NA=1** | 33 (14.2) | 21 (16.8) | 12 (11.2) | χ^2^ =2.58, df=1, p=0.28 |
| **Hypertension, n (%)** | 73 (31.5) | 52 (41.6) | 21 (19.6) | χ^2^ =11.9, df=1, p<0.001 |
| **Working Memory^a^, mean (SD) (NA=1)** | 0 (0.79) | 0.02 (0.77) | -0.02 (0.81) | F(1, 228)=2.18, p=0.14 |
| **Visual Construction^a^, mean (SD)** | 0 (0.8) | 0.05 (0.75) | -0.04 (0.85) | F(1, 229)=3.03, p=0.08 |
| **Episodic Memory^a^, mean (SD) (NA=3)** | 0.02 (0.8) | -0.17 (0.74) | 0.23 (0.81) | F(1, 226)=8.73, p=0.003 |
| **Executive Functions^a^, mean (SD) (NA=4)** | 0.01 (0.66) | 0 (0.61) | 0.02 (0.71) | F(1, 225)=0.93, p=0.34 |
| **Amyloid burden^a^, SUVR median [range]** | 1.22 [1.02 - 2.37] | 1.23 [1.02 - 2.23] | 1.19 [1.03 - 2.37] | F(1, 229)=0.87, p=0.35 |
| **Centiloid >12, n (%)** | 77 (33.2) | 51 (40.8) | 26 (24.3) | χ^2^ = 6.35, df = 1, p=0.01 |
| **Centiloid >30, n (%)** | 27 (11.6) | 15 (12.0) | 12 (11.2) | χ^2^ = 0, df = 1, p=1 |
| **WMH volume^b^ (ml), median [range] (NA=8)** | 2.5 [0.1, 40.9] | 2.8 [0.3, 29.7] | 2.2 [0.1, 40.9] | F(1, 220)=7.72, p=0.006 |
| **EC thickness^a^ (mm), mean (SD)** | 2.81 (0.27) | 2.84 (0.27) | 2.77 (0.27) | F(1, 229)=5.55, p=0.02 |
| **PhC thickness^a^ (mm), mean (SD)** | 2.47 (0.17) | 2.47 (0.17) | 2.48 (0.17) | F(1, 229)=0.11, p=0.74 |
| **Hippocampal volume^b^, mean (SD)** | 3.2 (0.36) | 3.3 (0.36) | 3.1 (0.32) | F(1, 228)=8.72, p=0.003 |
| **NEOcomp^a^ (mm), mean (SD)** | 2.29 (0.07) | 2.28 (0.07) | 2.3 (0.07) | F(1, 229)=0.07, p=0.79 |
| **Striatal volume^b^ (ml), mean (SD)** | 8.0 (0.92) | 8.4 (0.93) | 7.7 (0.75) | F(1, 228)=7.42, p=0.007 |
| **Lat. Ventricle volume^b^ (ml), median [range]** | 14.0 [4.6, 75.4] | 16.1 [5.2, 75.4] | 11.4 [4.6, 41.5] | F(1, 228)=3.91, p=0.049 |

Abbreviations: MCI = mild cognitive impairment; MMSE = Mini-Mental State Examination; BMI = body mass index; CVD = cardiovascular disease; SUVR = standardized uptake value ratio; WMH = white matter hyperintensity; EC = entorhinal cortex; PhC = parahippocampal cortex, NEOcomp = neocortical composite ROI

^a^Group differences were assessed by age-adjusted analysis of covariance (ANCOVA).

^b^Group differences were assessed by age- and total intracranial volume-adjusted ANCOVA.

**STable 2. Study cohort with amyloid-PET and tau PET scan: description overall and stratified by sex.**

|  | **ALL (n=93)** | **Men (n=54, 58.1%)** | **Women (n=39, 41.9%)** | **Group differences** |
| --- | --- | --- | --- | --- |
| **Age, years mean (SD) [range]** | 66.4 (7.9) | 67.0 (7.7) | 65.6 (8.2) | t = -0.82, df = 91, p = 0.41 |
| **Education, years mean (SD)** | 16.2 (2.8) | 17.2 (2.4) | 14.7 (2.6) | W = 517.5, p < 0.001 |
| **APOE-ε4 carriers, n (%)** | 19 (20.4) | 9 (16.7) | 10 (26.6) | χ^2^ = 0.64, df = 1, p = 0.42 |
| **MCI, n (%)** | 20 (21.5) | 16 (26.6) | 4 (10.2) | χ^2^ = 3.95, df = 1, p = 0.047 |
| **MMSE, mean (SD)** | 29.1 (1.1) | 29.0 (1.1) | 29.3 (1.0) | t = 1.37, df = 91, p = 0.17 |
| **BMI, mean (SD)** | 25.5 (4.0) | 26.3 (3.3) | 24.3 (4.7) | t = -2.41, df = 91, p = 0.02 |
| **Diabetes mellitus, n (%)** | 4 (4.3) | 3 (5.6) | 1 (2.6) | χ^2^ = 0.03, df = 1, p = 0.85 |
| **CVD, n (%)** | 12 (12.9) | 8 (14.8) | 4 (10.2) | χ^2^ = 4.98, df = 1, p = 0.03 |
| **Hypertension, n (%)** | 27 (29.0) | 21 (38.9) | 6 (15.4) | χ^2^ = 0.11, df = 1, p = 0.74 |
| **Working Memory** | 0.13 (0.84) | 0.18 (0.77) | 0.07 (0.93) | t = -0.59, df = 91, p = 0.56 |
| **Visual Construction** | 0.12 (0.74) | 0.1 (0.64) | 0.13 (0.35) | t = 0.18, df = 91, p = 0.85 |
| **Episodic Memory (NA=2)** | 0.09 (0.73) | -0.07 (0.76) | 0.31 (0.64) | t = 2.52, df = 89, p = 0.01 |
| **Executive Functions (NA=1)** | 0.06 (0.62) | 0.08 (0.61) | 0.04 (0.65) | t = -0.31, df = 90, p = 0.76 |
| **Amyloid burden, SUVR median [range]** | 1.22 [1.02; 2.14] | 1.24 [1.02; 2.14] | 1.19 [1.07; 2.00] | t = -1.13, df = 91, p = 0.26 |
| **Centiloid >12, n (%)** | 28 (30.1) | 22 (40.7) | 6 (15.4) | χ^2^ = 5.77, df = 1, p = 0.02 |
| **Centiloid >30, n (%)** | 8 (8.6) | 6 (11.1) | 2 (5.1) | χ^2^ = 0.41, df = 1, p = 0.52 |
| **MTL tau, SUVR median [range]** | 1.1 [0.85, 1.61] | 1.07 [0.85, 1.55] | 1.12 [0.92, 1.61] | t = 2.67, df = 91, p = 0.009 |
| **NEO tau, SUVR median [range]** | 1.12 [0.92, 1.8] | 1.08 [0.92, 1.27] | 1.19 [1.01, 1.8] | t = 2.67, df = 91, p = 0.009 |
| **WMH volume^a^ (ml), median [range]** | 2.6 [0.1, 29.8] | 2.7 [0.3; 17.4] | 2.5 [0.1, 29.8] | F(1, 85 )= 5.00, p = 0.04 |
| **EC thickness (mm), mean (SD)** | 2.84 (0.26) | 2.89 (0.26) | 2.77 (0.24) | t = -2.26, df = 91, p = 0.03 |
| **PhC thickness (mm), mean (SD)** | 2.49 (0.15) | 2.5 (0.14) | 2.48 (0.15) | t = -0.57, df = 91, p = 0.57 |
| **Hippocampal volume^a^, mean (SD)** | 3.2 (0.34) | 3.3 (0.34) | 3.0 (0.37) | F(1, 90) = 0.05, p = 0.83 |
| **NEO comp. (mm), mean (SD)** | 2.29 (0.07) | 2.29 (0.07) | 2.29 (0.07) | t = 0, df = 91, p = 1 |
| **Striatal volume^a^ (ml), mean (SD)** | 8.1 (0.96) | 8.4 (0.96) | 7.7 (0.78) | F(1, 90) = 2.19, p = 0.14 |
| **Lat. Ventricle volume^a^ (ml), median [range]** | 15.4 [5.2, 40.2] | 18.3 [6.8, 40.2] | 13.9 [5.3, 34.0] | F(1, 90) = 0, p = 0.95 |

Abbreviations: MCI = mild cognitive impairment; MMSE = Mini-Mental State Examination; BMI = body mass index; CVD = cardiovascular disease; SUVR = standardized uptake value ratio; WMH = white matter hyperintensity; EC = entorhinal cortex; PhC = parahippocampal cortex, NEOcomp = neocortical composite ROI

^a^Group differences were assessed by total intracranial volume-adjusted ANCOVA.

**STable 3. Structural equation model parameter estimates for total cohort and CU participants with episodic memory and executive function as outcome variables.**

|  |  | **Total cohort** | | **CU only** | |
| --- | --- | --- | --- | --- | --- |
| **Latent variables** | **derived from** | **β (95% CI)** | ***P*-value** | **β (95% CI)** | ***P*-value** |
| **MEM** |  |  |  |  |  |
|  | CERAD learning (word) | 0.77 (0.69 to 0.85) | **<0.001** | 0.697 (0.552 to 0.841) | **<0.001** |
|  | RAVLT trial 5 | 0.79 (0.73 to 0.85) | **<0.001** | 0.688 (0.561 to 0.815) | **<0.001** |
|  | RAVLT late recall (word) | 0.79 (0.70 to 0.87) | **<0.001** | 0.65 (0.477 to 0.824) | **<0.001** |
|  | CERAD recognition | 0.86 (0.79 to 0.93) | **<0.001** | 0.742 (0.622 to 0.863) | **<0.001** |
|  | RAVLT recognition | 0.69 (0.56 to 0.82) | **<0.001** | 0.395 (0.147 to 0.643) | **0.001** |
|  | CERAD recall (word) | 0.86 (0.79 to 0.93) | **<0.001** | 0.744 (0.618 to 0.869) | **<0.001** |
|  | CERAD recall (figure) | 0.42 (0.24 to 0.61) | **<0.001** | 0.212 (0.018 to 0.406) | **0.046** |
| **EXE** |  |  |  |  |  |
|  | Category fluency | 0.51 (0.39 to 0.63) | **<0.001** | 0.531 (0.37 to 0.691) | **<0.001** |
|  | Letter fluency (s) | 0.44 (0.33 to 0.56) | **<0.001** | 0.425 (0.266 to 0.583) | **<0.001** |
|  | Stroop test | 0.64 (0.55 to 0.73) | **<0.001** | 0.616 (0.491 to 0.742) | **<0.001** |
|  | Trail Making Test | 0.42 (0.27 to 0.57) | **<0.001** | 0.215 (0.025 to 0.405) | **0.038** |
|  | Figural fluency | 0.67 (0.57 to 0.77) | **<0.001** | 0.571 (0.406 to 0.736) | **<0.001** |
| **Regressions** | **regressed on** | **β (95% CI)** | ***P*-value** | **β (95% CI)** | ***P*-value** |
| **MEM** |  |  |  |  |  |
|  | EC thickness | 0.075 (-0.056 to 0.207) | 0.256 | 0.068 (-0.105 to 0.241) | 0.438 |
|  | PhC thickness | 0.129 (0.009 to 0.249) | **0.036** | 0.068 (-0.122 to 0.258) | 0.491 |
|  | NEOcomp thickness | -0.215 (-0.359 to -0.07) | **0.005** | -0.199 (-0.404 to 0.006) | 0.067 |
|  | Hippocampal volume | 0.152 (-0.029 to 0.332) | 0.1 | 0.17 (-0.065 to 0.404) | 0.18 |
|  | Striatal volume | -0.102 (-0.261 to 0.057) | 0.223 | -0.094 (-0.326 to 0.138) | 0.45 |
|  | Lat. ventricle volume | -0.144 (-0.353 to 0.066) | 0.19 | -0.057 (-0.295 to 0.181) | 0.64 |
|  | WMH volume | -0.048 (-0.261 to 0.165) | 0.66 | 0.014 (-0.113 to 0.393) | 0.279 |
|  | Amyloid burden | -0.156 (-0.332 to 0.019) | 0.074 | 0.085 (-0.062 to 0.232) | 0.264 |
|  | Age | -0.287 (-0.463 to -0.111) | **0.001** | -0.32 (-0.538 to -0.103) | **0.005** |
|  | total ICV | -0.04 (-0.273 to 0.194) | 0.739 | -0.047 (-0.302 to 0.208) | 0.719 |
|  | Sex | -0.276 (-0.428 to -0.123) | **<0.001** | -0.428 (-0.595 to -0.262) | **<0.001** |
|  | Years of Education | 0.226 (0.1 to 0.352) | **0.001** | 0.209 (0.041 to 0.377) | **0.022** |
|  | APOE4 | 0.026 (-0.087 to 0.139) | 0.647 | -0.009 (-0.162 to 0.143) | 0.903 |
| **EXE** |  |  |  |  |  |
|  | EC thickness | 0.019 (-0.119 to 0.157) | 0.786 | 0.004 (-0.172 to 0.181) | 0.963 |
|  | PhC thickness | -0.06 (-0.221 to 0.102) | 0.476 | -0.107 (-0.318 to 0.105) | 0.341 |
|  | NEOcomp thickness | -0.008 (-0.15 to 0.133) | 0.907 | 0.042 (-0.141 to 0.226) | 0.652 |
|  | Hippocampal volume | -0.033 (-0.24 to 0.173) | 0.748 | -0.088 (-0.322 to 0.146) | 0.452 |
|  | Striatal volume | 0.139 (-0.034 to 0.312) | 0.107 | 0.155 (-0.043 to 0.354) | 0.131 |
|  | Lat. ventricle volume | -0.349 (-0.557 to -0.14) | **0.001** | -0.31 (-0.56 to -0.06) | **0.021** |
|  | WMH volume | 0.035 (-0.131 to 0.2) | 0.412 | -0.025 (-0.367 to 0.317) | 0.885 |
|  | Amyloid burden | -0.065 (-0.239 to 0.108) | 0.449 | 0.088 (-0.097 to 0.273) | 0.347 |
|  | Age | -0.35 (-0.536 to -0.164) | **<0.001** | -0.326 (-0.543 to -0.11) | **0.004** |
|  | total ICV | 0.213 (-0.01 to 0.437) | 0.055 | 0.285 (0.038 to 0.533) | **0.021** |
|  | Sex | -0.14 (-0.295 to 0.016) | 0.093 | -0.175 (-0.371 to 0.021) | 0.097 |
|  | Years of Education | 0.353 (0.219 to 0.488) | **<0.001** | 0.332 (0.159 to 0.505) | **0.004** |
|  | APOE4 | 0.04 (-0.08 to 0.16) | 0.516 | 0.02 (-0.135 to 0.175) | 0.799 |
| **EC thickness** |  |  |  |  |  |
|  | Amyloid burden | -0.163 (-0.293 to -0.033) | **0.01** | -0.025 (-0.162 to 0.112) | 0.722 |
|  | Age | -0.146 (-0.281 to -0.01) | **0.04** | -0.129 (-0.285 to 0.026) | 0.106 |
|  | Sex | 0.146 (0.02 to 0.272) | **0.024** | 0.147 (0 to 0.294) | 0.051 |
|  | APOE4 | 0.021 (-0.103 to 0.145) | 0.745 | -0.016 (-0.165 to 0.133) | 0.835 |
| **PhC thickness** |  |  |  |  |  |
|  | Amyloid burden | -0.122 (-0.277 to 0.033) | 0.119 | -0.041 (-0.21 to 0.128) | 0.623 |
|  | Age | -0.127 (-0.257 to 0.003) | 0.059 | -0.119 (-0.267 to 0.028) | 0.114 |
|  | Sex | -0.03 (-0.156 to 0.096) | 0.642 | 0.024 (-0.123 to 0.171) | 0.749 |
|  | APOE4 | -0.008 (-0.135 to 0.119) | 0.903 | -0.037 (-0.181 to 0.106) | 0.613 |
| **NEOcomp thickness** |  |  |  |  |  |
|  | Amyloid burden | -0.26 (-0.406 to -0.113) | **0.001** | -0.218 (-0.371 to -0.065) | **0.006** |
|  | Age | -0.304 (-0.417 to -0.191) | **<0.001** | -0.319 (-0.441 to -0.198) | **<0.001** |
|  | Sex | -0.025 (-0.138 to 0.088) | 0.661 | -0.027 (-0.163 to 0.108) | 0.692 |
|  | APOE4 | 0.07 (-0.048 to 0.188) | 0.249 | 0.057 (-0.078 to 0.192) | 0.412 |
| **Hippocamal volume** |  |  |  |  |  |
|  | Amyloid burden | -0.097 (-0.185 to -0.009) | **0.02** | -0.092 (-0.197 to 0.013) | 0.07 |
|  | Age | -0.383 (-0.493 to -0.272) | **<0.001** | -0.363 (-0.503 to -0.222) | **<0.001** |
|  | total ICV | 0.412 (0.142 to 0.683) | **0.004** | 0.385 (0.061 to 0.709) | **0.025** |
|  | Sex | 0.185 (0.013 to 0.357) | **0.033** | 0.224 (0.03 to 0.418) | **0.023** |
|  | APOE4 | 0.057 (-0.03 to 0.143) | 0.198 | 0.04 (-0.062 to 0.142) | 0.443 |
| **Striatal volume** |  |  |  |  |  |
|  | Amyloid burden | -0.032 (-0.14 to 0.076) | 0.563 | -0.092 (-0.22 to 0.037) | 0.164 |
|  | Age | -0.141 (-0.265 to -0.016) | **0.021** | -0.111 (-0.273 to 0.05) | 0.16 |
|  | total ICV | 0.19 (0.012 to 0.368) | **0.033** | 0.22 (0.04 to 0.4) | **0.016** |
|  | Sex | 0.383 (0.129 to 0.637) | **0.006** | 0.301 (0.045 to 0.557) | **0.027** |
|  | APOE4 | 0.046 (-0.07 to 0.162) | 0.44 | 0.048 (-0.085 to 0.181) | 0.476 |
| **Lat. ventricle volume** |  |  |  |  |  |
|  | Age | 0.434 (0.35 to 0.517) | **<0.001** | 0.377 (0.272 to 0.482) | **<0.001** |
|  | total ICV | 0.611 (0.482 to 0.74) | **<0.001** | 0.624 (0.442 to 0.807) | **<0.001** |
|  | Sex | -0.113 (-0.24 to 0.015) | 0.093 | -0.108 (-0.269 to 0.054) | 0.219 |
| **WMH volume** |  |  |  |  |  |
|  | Amyloid burden | 0.047 (-0.079 to 0.173) | 0.462 | -0.148 (-0.252 to -0.043) | **0.006** |
|  | Age | 0.481 (0.398 to 0.564) | **<0.001** | 0.547 (0.462 to 0.632) | **<0.001** |
|  | total ICV | 0.226 (0.072 to 0.38) | **0.004** | 0.36 (0.205 to 0.514) | **<0.001** |
|  | Sex | -0.201 (-0.329 to 0.074) | **0.002** | -0.163 (-0.325 to -0.002) | **0.047** |
|  | APOE4 | -0.078 (-0.156 to 0) | 0.051 | -0.072 (-0.151 to 0.008) | 0.076 |
| **Amyloid burden** |  |  |  |  |  |
|  | Age | 0.4 (0.309 to 0.49) | **<0.001** | 0.325 (0.223 to 0.426) | **<0.001** |
|  | Sex | -0.036 (-0.153 to 0.08) | 0.547 | 0.073 (-0.074 to 0.22) | 0.296 |
|  | APOE4 | 0.242 (0.121 to 0.364) | **<0.001** | 0.229 (0.093 to 0.366) | **0.01** |

The significant association between higher WMH volume and lower amyloid burden in CU participants is contrary to what we expected. We interpret this finding as a possible synergistic effect of WMH and amyloid burden. A participant with a large WMH volume would have to have low amyloid pathology (or vice versa) to be cognitively unimpaired. Parameter estimates are standardized. “Sex” was coded as women = 0 and men = 1. We used the German version of the RAVLT (“VLMT”). Abbreviations: MEM = episodic memory; EXE = executive function; EC = entorhinal cortex; PhC = parahippocampal cortex; ICV = intracranial volume; WMH = white matter hyperintensity.

**STable 4. Structural equation model parameter estimates for total cohort and CU participants with global cognition as outcome variables.**

|  |  | **Total cohort** | | **CU only** | |
| --- | --- | --- | --- | --- | --- |
| **Latent variables** | **derived from** | **β (95% CI)** | ***P*-value** | **β (95% CI)** | ***P*-value** |
| **Global Cognition** |  |  |  |  |  |
|  | WM | 0.641 (0.553 to 0.73) | **<0.001** | 0.559 (0.439 to 0.678) | **<0.001** |
|  | EXE | 0.787 (0.7 to 0.88) | **<0.001** | 0.774 (0.638 to 0.91) | **<0.001** |
|  | MEM | 0.661 (0.53 to 0.79) | **<0.001** | 0.564 (0.396 to 0.731) | **<0.001** |
|  | VC | 0.486 (0.36 to 0.62) | **<0.001** | 0.18 (0.007to 0.353) | **0.041** |
| **Regressions** | **regressed on** | **β (95% CI)** | ***P*-value** | **β (95% CI)** | ***P*-value** |
| **Global Cognition** |  |  |  |  |  |
|  | EC thickness | 0.054 (-0.071 to 0.179) | 0.399 | 0.077 (-0.079 to 0.232) | 0.334 |
|  | PhC thickness | 0 (-0.125 to 0.126) | 0.996 | -0.043 (-0.216 to 0.13) | 0.628 |
|  | NEOcomp thickness | -0.059 (-0.195 to 0.077) | 0.394 | 0.022 (-0.172 to 0.215) | 0.826 |
|  | Hippocampal volume | 0.027 (-0.156 to 0.211) | 0.769 | -0.009 (-0.254 to 0.236) | 0.946 |
|  | Striatal volume | 0.063 (-0.105 to 0.23) | 0.462 | 0.068 (-0.137 to 0.273) | 0.514 |
|  | Lat. ventricle volume | -0.34 (-0.555 to -0.125) | **0.002** | -0.298 (-0.532 to -0.064) | **0.013** |
|  | WMH volume | -0.067 (-0.271 to 0.136) | 0.515 | 0.119 (-0.105 to 0.343) | 0.299 |
|  | Amyloid burden | -0.14 (-0.322 to 0.042) | 0.13 | 0.094 (-0.07 to 0.258) | 0.263 |
|  | Age | -0.284 (-0.448 to -0.12) | **0.001** | -0.335 (-0.535 to -0.135) | **0.001** |
|  | total ICV | 0.223 (0.002 to 0.445) | **0.048** | 0.244 (-0.002 to 0.489) | 0.052 |
|  | Sex | -0.218 (-0.367 to -0.068) | **0.004** | -0.289 (-0.499 to -0.079) | **0.007** |
|  | Years of Education | 0.354 (0.226 to 0.482) | **<0.001** | 0.361 (0.207 to 0.519) | **<0.001** |
|  | APOE4 | 0.01 (-0.107 to 0.127) | 0.867 | 0.014 (-0.148 to 0.175) | 0.868 |

Only estimates of paths going to “Global Cognition” are shown as the remaining estimates are practically identical to the estimates reported in Supplementary Table 3. Parameter estimates are standardized. “Sex” was coded as women = 0 and men = 1. Abbreviations: WM = working memory; EXE = executive function; MEM = episodic memory; VC = visual construction; EC = entorhinal cortex; PhC = parahippocampal cortex; ICV = intracranial volume; WMH = white matter hyperintensity.

**Multigroup analysis**

In structural equation modeling (SEM), the examination of differences and similarities between groups becomes more informative and reliable when the underlying latent structure of the investigated latent constructs (in our case the episodic memory and executive function constructs) is the same for males and females, that is, the constructs are invariant and measurement invariance (MI) is established. If MI is not supported, the comparison between groups is compromised because the meaning of the underlying construct becomes group specific. Establishing MI allowed us to compare the structural parameters between men and women. MI of the scale’s items across sex groups was tested based on published guidelines for establishing MI of models (2, 3).

**STable 5. Fit indices for measurement invariance testing procedure women vs. men.**

| **#** | **Model** | **df** | **χ2** | **p-value** | **SRMR** | **RMSEA (90% CI)** | **TLI** | **CFI** | **AIC** | **BIC** | **Remark** | |
| --- | --- | --- | --- | --- | --- | --- | --- | --- | --- | --- | --- | --- |
| **1** | Complete Baseline model, total cohort | 67 | 121.007 | <0.001 | 0.051 | 0.059 (0.042, 0.076) | 0.967 | 0.971 | 6062.271 | 6224.26 | FIML estimator | |
|  |  | | | | | | | | | | | |
| **#** | **Model** | **df** | **χ2** | **p-value** | **SRMR** | **RMSEA (90% CI)** | **TLI** | **CFI** | **AIC** | **BIC** | **Remark** | |
| **2** | Men only | 67 | 76.393 | 0.202 | 0.050 | 0.034 (0.00, 0.065) | 0.985 | 0.989 | 3166.236 | 3264.376 | Total: n=125; Used: n=122 | |
| **3** | Women only | 67 | 89.051 | 0.037 | 0.055 | 0.054 (0.014, 0.082) | 0.968 | 0.976 | 2650.756 | 2743.645 | Total: n=107; Used: n=105 | |
|  |  | | | | | | | | | | | |
| **#** | **Model** | **df** | **χ2** | **p-value** | **SRMR** | **RMSEA (90% CI)** | **TLI** | **CFI** | **AIC** | **BIC** | **Δχ^2^ p-value** | **Comparison** |
| **4** | configural invariance | 134 | 165.088 | 0.035 | 0.053 | 0.047 (0.018, 0.067) | 0.976 | 0.982 | 5867.676 | 6175.922 |  |  |
| **5** | metric invariance (factor loadings constr.) | 146 | 184.654 | 0.017 | 0.077 | 0.048 (0.022, 0.069) | 0.972 | 0.977 | 5864.681 | 6145.527 | 0.1022 | 4 vs 5 |
| **6** | scalar invariance (intercepts const.) | 158 | 236.414 | <0.001 | 0.104 | 0.066 (0.048, 0.083) | 0.948 | 0.954 | 5891.447 | 6131.193 | <0.001 | 5 vs 6 |
|  | *separate model for each construct* |  |  |  |  |  |  |  |  |  |  |  |
| **7a** | intercepts of EF indicators const. | 151 | 192.221 | 0.013 | 0.078 | 0.049 (0.024, 0.068) | 0.971 | 0.976 | 5862.256 | 6125.977 | 0.1829 | 5 vs. 7a |
| **7b** | intercepts of EM indicators const. | 153 | 227.165 | <0.001 | 0.104 | 0.065 (0.046, 0.082) | 0.949 | 0.957 | 5892.387 | 6149.258 | <0.001 | 5 vs. 7b |
|  | *separate model for each EM indicator* |  |  |  |  |  |  |  |  |  |  |  |
| **7b.1** | CERAD learning const. | 147 | 210.521 | <0.001 | 0.095 | 0.061 (0.041, 0.079) | 0.954 | 0.963 | 5888.283 | 6165.704 | **<0.001** | 5 vs. 7b.1 |
| **7b.2** | RAVLT trial 5 const. | 147 | 200.432 | 0.002 | 0.089 | 0.056 (0.035, 0.075) | 0.962 | 0.969 | 5878.300 | 6155.721 | **<0.001** | 5 vs. 7b.2 |
| **7b.3** | RAVLT late recall const. | 147 | 204.222 | 0.001 | 0.093 | 0.058 (0.037, 0.077) | 0.959 | 0.966 | 5882.018 | 6159.439 | **<0.001** | 5 vs. 7b.3 |
| **7b.4** | CERAD recognition const. | 147 | 203.851 | 0.001 | 0.094 | 0.058 (0.037, 0.076) | 0.959 | 0.967 | 5881.949 | 6159.370 | **<0.001** | 5 vs. 7b.4 |
| **7b.5** | RAVLT recognition const. | 147 | 190.690 | 0.009 | 0.081 | 0.051 (0.027, 0.070) | 0.969 | 0.974 | 5868.750 | 6146.171 | **0.01708** | 5 vs. 7b.5 |
| **7b.6** | CERAD recall (word) const. | 147 | 206.950 | 0.001 | 0.096 | 0.06 (0.039, 0.078) | 0.957 | 0.965 | 5884.813 | 6162.234 | **<0.001** | 5 vs. 7b.6 |
| **7b.7** | CERAD recall (figure) const. | 147 | 186.223 | 0.016 | 0.077 | 0.048 (0.022, 0.068) | 0.972 | 0.977 | 5865.894 | 6129.615 | 0.2038 | 5 vs. 7b.7 |
| **8** | partial scalar invariance | 152 | 194.006 | 0.012 | 0.079 | 0.049 (0.024, 0.068) | 0.971 | 0.975 | 5861.816 | 6122.112 | 0.1535 | 5 vs. 8 |
| **9** | residual variance invariance | 164 | 205.585 | 0.015 | 0.087 | 0.048 (0.022, 0.067) | 0.973 | 0.972 | 5862.026 | 6077.798 | 0.3914 | 8 vs. 9 |
|  | *invariance of residual covariances* |  |  |  |  |  |  |  |  |  |  |  |
| **10** | Residual covariances const. | 170 | 215.433 | 0.010 | 0.087 | 0.050 (0.026, 0.069) | 0.970 | 0.971 | 5862.994 | 6061.641 | 0.193 | 9 vs. 10 |
|  |  | | | | | | | | | | | |
| **#** | **Model** | **df** | **χ2** | **p-value** | **SRMR** | **RMSEA (90% CI)** | **TLI** | **CFI** | **AIC** | **BIC** | **Δχ^2^ p-value** | **Comparison** |
| **11** | partial scalar invariance + residual invariance | 166 | 209.831 | 0.012 | 0.079 | 0.050 (0.025, 0.069) | 0.970 | 0.972 | 5865.468 | 6077.815 |  |  |
| **12** | factor variance + covariance invariance | 169 | 215.042 | 0.01 | 0.087 | 0.051 (0.026, 0.07) | 0.969 | 0.971 | 5865.320 | 6067.392 | 0.1637 | 11 vs 12 |
|  |  | | | | | | | | | | | |
| **#** | **Model** | **df** | **χ2** | **p-value** | **SRMR** | **RMSEA (90% CI)** | **TLI** | **CFI** | **AIC** | **BIC** | **Remark** | |
| **13** | Final multigroup model | 169 | 226.231 | 0.002 | 0.088 | 0.054 (0.035, 0.074) | 0.965 | 0.967 | 6036.742 | 6240.099 | FIML estimator | |

Model #4-10: all factor variances = 1 and all factor intercepts = 0; model #11-13: varying factor variances and varying factor intercepts. The measurement invariance procedure was conducted in a model that included the EM and EF constructs controlled for age and years of education. No other variables were included in the model. The testing procedure was conducted only with cases that had all data available using robust maximum likelihood estimator. We used the χ^2^ difference test to determine invariance between the less restrictive model and more restrictive model. A p-value <0.05 indicated that the hypothesis of invariance should be rejected. For instance, model #7b showed a significant lower fit to data compared to model #6, indicating that constraining the intercepts of the indicators of the EM construct to be equal across sex decreased the model fit to data. Therefore, the intercept of each indicator was separately tested. If the constraint that the intercepts be the same for women and men resulted in a significant degradation of the model fit to the data, the estimate of the intercept for the indicator was allowed to vary by sex. The p-values for such indicators are in bold. Abbreviations: const. = constrained; FIML = full information maximum likelihood, df = degrees of freedom; SRMR = standardized root mean square residual; RMSEA = root mean square error of approximation; TLI = Tucker-Lewis index; CFI = comparative fit index; AIC = Akaike information criterion: BIC = Bayesian information criterion; CI = confidence interval.

**STable 6. Structural equation model parameter estimates for men and women in executive function sub-model.**

|  |  | **Men** | | **Women** | |
| --- | --- | --- | --- | --- | --- |
| **Regressions** | **regressed on** | **β (95% CI)** | ***P*-value** | **β (95% CI)** | ***P*-value** |
| **EXE** |  |  |  |  |  |
|  | NEOcomp thickness | 0.022 (-0.08 to 0.123) | 0.672 | -0.026 (-0.129 to 0.076) | 0.61 |
|  | Striatal volume | 0.049 (-0.045 to 0.143) | 0.09 | 0.05 (-0.07 to 0.17) | 0.417 |
|  | WMH volume | 0.071 (-0.049 to 0.191) | 0.248 | -0.02 (-0.097 to 0.057) | 0.604 |
|  | Lat. ventricle volume | -0.146 (-0.268 to -0.025) | **0.018** | -0.164 (-0.302 to -0.026) | **0.02** |
|  | Age | -0.134 (-0.255 to -0.013) | **0.03** | -0.216 (-0.35 to -0.082) | **0.002** |
|  | Years of Education | 0.192 (0.086 to 0.298) | **<0.001** | 0.138 (0.034 to 0.243) | **0.009** |
|  | total ICV | 0.033 (-0.093 to 0.159) | 0.603 | 0.215 (0.058 to 0.373) | **0.007** |
| **NEOcomp thickness** |  |  |  |  |  |
|  | Age | -0.433 (-0.607 to -0.258) | **<0.001** | -0.385 (-0.557 to -0.214) | **<0.001** |
| **Striatal volume** |  |  |  |  |  |
|  | Age | -0.179 (-0.36 to 0.002) | 0.052 | -0.111 (-0.238 to 0.017) | 0.088 |
|  | total ICV | 0.326 (0.003 to 0.649) | **0.048** | 0.536 (0.301 to 0.775) | **<0.001** |
| **WMH volume** |  |  |  |  |  |
|  | Age | 0.412 (0.264 to 0.561) | **<0.001** | 0.615 (0.398 to 0.832) | **<0.001** |
|  | total ICV | 0.17 (0.001 to 0.339) | **0.049** | 0.371 (0.099 to 0.644) | **0.008** |
| **Lat. ventricle volume** |  |  |  |  |  |
|  | Age | 0.436 (0.274 to 0.598) | **<0.001** | 0.399 (0.276 to 0.522) | **<0.001** |
|  | total ICV | 0.69 (0.503 to 0.876) | **<0.001** | 0.433 (0.248 to 0.623) | **<0.001** |

Model parameter estimates for both models are unstandardized so that estimates for the same path can be compared across models. Abbreviations: WM = working memory; EXE = executive function; ICV = intracranial volume; WMH = white matter hyperintensity

**STable 7. Structural equation model parameter estimates for men and women in episodic memory sub-model.**

| **Regressions** |  | **Men** | | **Women** | |
| --- | --- | --- | --- | --- | --- |
|  | **regressed on** | **β (95% CI)** | ***P*-value** | **β (95% CI)** | ***P*-value** |
| **MEM** |  |  |  |  |  |
|  | EC thickness | 0.129 (-0.013 to 0.27) | 0.074 | -0.042 (-0.159 to 0.076) | 0.488 |
|  | PhC thickness | 0.154 (0.033 to 0.275) | **0.013** | 0.051 (-0.069 to 0.171) | 0.405 |
|  | NEOcomp thickness | -0.208 (-0.347 to -0.069) | **0.003** | -0.092 (-0.25 to 0.066) | 0.253 |
|  | Hippocamal volume | 0.122 (-0.055 to 0.299) | 0.175 | 0.044 (-0.136 to 0.224) | 0.634 |
|  | Amyloid burden | -0.016 (-0.163 to 0.13) | 0.827 | -0.234 (-0.414 to -0.054) | **0.011** |
|  | Age | -0.2 (-0.378 to -0.022) | **0.028** | -0.305 (-0.455 to -0.155) | **<0.001** |
|  | total ICV | -0.191 (-0.378 to -0.004) | **0.045** | 0.013 (-0.21 to 0.236) | 0.91 |
|  | Years of Education | 0.139 (-0.002 to 0.281) | 0.054 | 0.152 (0.022 to 0.281) | **0.022** |
|  | APOE4 | 0.001 (-0.274 to 0.277) | 0.992 | 0.126 (-0.136 to 0.387) | 0.347 |
| **EC thickness** |  |  |  |  |  |
|  | Amyloid burden | -0.163 (-0.351 to 0.026) | 0.091 | -0.161 (-0.322 to 0) | **0.05** |
|  | Age | -0.068 (-0.263 to 0.126) | 0.49 | -0.218 (-0.412 to -0.024) | **0.028** |
|  | APOE4 | 0.08 (-0.347 to 0.506) | 0.714 | 0.031 (-0.383 to 0.446) | 0.882 |
| **PhC thickness** |  |  |  |  |  |
|  | Amyloid burden | 0.017 (-0.268 to 0.303) | 0.905 | -0.239 (-0.401 to -0.076) | **0.004** |
|  | Age | -0.204 (-0.399 to -0.01) | **0.04** | -0.048 (-0.218 to 0.121) | 0.575 |
|  | APOE4 | 0.175 (-0.216 to 0.566) | 0.381 | -0.158 (-0.529 to 0.212) | 0.402 |
| **NEOcomp thickness** |  |  |  |  |  |
|  | Amyloid burden | -0.292 (-0.575 to -0.009) | **0.043** | -0.238 (-0.384 to -0.092) | **0.001** |
|  | Age | -0.332 (-0.5 to -0.163) | **<0.001** | -0.287 (-0.453 to -0.122) | **0.001** |
|  | APOE4 | 0.285 (-0.058 to 0.629) | 0.104 | 0.024 (-0.345 to 0.393) | 0.9 |
| **Hippocampal volume** |  |  |  |  |  |
|  | Amyloid burden | -0.115 (-0.287 to 0.056) | 0.188 | -0.113 (-0.196 to -0.03) | **0.007** |
|  | Age | -0.396 (-0.591 to -0.201) | **<0.001** | -0.305 (-0.429 to -0.181) | **<0.001** |
|  | total ICV | 0.348 (0.02 to 0.676) | **0.038** | 0.597 (0.402 to 0.791) | **<0.001** |
|  | APOE4 | 0.379 (0.051 to 0.707) | **0.023** | -0.176 (-0.421 to 0.07) | 0.16 |
| **Amyloid burden** |  |  |  |  |  |
|  | Age | 0.322 (0.163 to 0.481) | **<0.001** | 0.452 (0.268 to 0.636) | **<0.001** |
|  | APOE4 | 0.279 (0.047 to 0.511) | **0.018** | 0.791 (0.287 to 1.295) | **0.002** |

Model parameter estimates for both models are unstandardized so that estimates for the same path can be compared across models. Abbreviations: MEM = episodic memory; EC = entorhinal cortex; PhC = parahippocampal cortex; ICV = intracranial volume


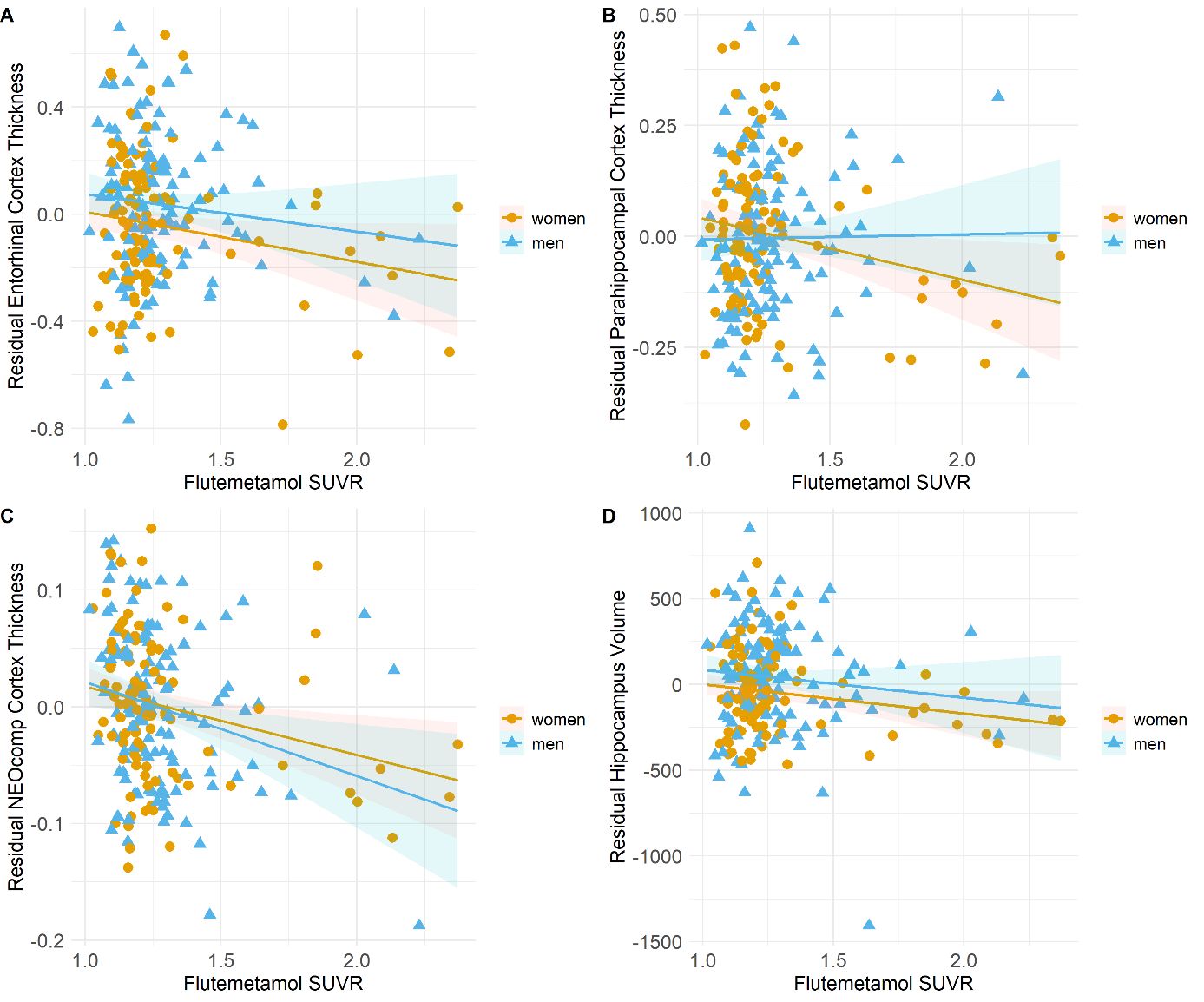


**SFigure 2. Correlation plots demonstrating associations between Aβ burden and cortical thickness/volume of the investigated ROIs.** The values in the graphs are the residuals of entorhinal cortex thickness (A), parahippocampal cortex thickness (B), NEOcomp cortex thickness (C), and hippocampal volume (D) after regressing out the effects of age and APOE4 and, in the case of hippocampal volume, additionally of total intracranial volume. Abbreviations: SUVR = standardized uptake value ratio.

| **Regressions** |  | **CL >12** | | **Continuous amyloid** | |
| --- | --- | --- | --- | --- | --- |
|  | **regressed on** | **β (95% CI)** | ***P*-value** | **β (95% CI)** | ***P*-value** |
| **MEM** |  |  |  |  |  |
|  | EC thickness | 0.037 (-0.122 to 0.197) | 0.646 |  |  |
|  | PhC thickness | 0.105 (-0.035 to 0.246) | 0.142 |  |  |
|  | Hippocamal volume | 0.243 (0.001 to 0.488) | **0.049** |  |  |
|  | NEOcomp thickness | -0.037 (-0.172 to 0.098) | 0.591 |  |  |
|  | MTL tau | -0.119 (-0.309 to 0.07) | 0.218 |  |  |
|  | Age | -0.056 (-0.210 to 0.098) | 0.479 |  |  |
|  | Years of Education | 0.136 (-0.013 to 0.285) | 0.074 |  |  |
|  | Sex | -0.683 (-1.094 to -0.272) | 0.001 |  |  |
|  | total ICV | -0.108 (-0.314 to 0.098) | 0.303 |  |  |
|  | time diff. PET scans | 0.023 (-0.104 to 149) | 0.723 |  |  |
| **EC thickness** |  |  |  |  |  |
|  | MTL tau | -0.327 (-0.534 to -0.119) | **0.002** |  |  |
|  | Age | -0.051 (-0.224 to 0.122) | 0.565 |  |  |
|  | Sex | 0.295 (-0.085 to 0.676) | 0.128 |  |  |
| **PhC thickness** |  |  |  |  |  |
|  | MTL tau | -0.204 (-0.515 to 0.107) | 0.199 |  |  |
|  | Age | -0.07 (-0.274 to 0.134) | 0.5 |  |  |
|  | Sex | 0.022 (-0.431 to 0.475) | 0.924 |  |  |
| **Hippocampal volume** |  |  |  |  |  |
|  | MTL tau | -0.284 (-0.388 to -0.18) | **<0.001** |  |  |
|  | Age | -0.228 (-0.354 to -0.103) | **<0.001** |  |  |
|  | Sex | -0.024 (-0.372 to 0.323) | 0.89 |  |  |
|  | total ICV | 0.611 (0.428 to 0.795) | **<0.001** |  |  |
| **NEOcomp thickness** |  |  |  |  |  |
|  | NEO tau | -0.162 (-0.345 to 0.02) | 0.081 |  |  |
|  | Age | -0.317 (-0.497 to -0.138) | **0.001** |  |  |
|  | Sex | -0.076 (-0.492 to 0.34) | 0.72 |  |  |
| **MTL tau** |  |  |  |  |  |
|  | Amyloid burden | 0.086 (-0.51 to 0.682) | 0.778 | 0.494 (0.172 to 0.816) | **0.003** |
|  | Age | 0.266 (0.061 to 0.472) | **0.011** | 0.056 (-0.153 to 0.265) | 0.598 |
|  | Sex | -0.588 (-1.032 to -0.143) | **0.01** | -0.66 (-0.995 to -0.325) | **<0.001** |
|  | time diff. PET scans | -0.03 (-0.22 to 0.16) | 0.757 | -0.067 (-0.223 to 0.09) | 0.403 |
|  | APOE4 | 0.23 (-0.184 to 0.645) | 0.276 | 0.056 (-0.264 to 0.377) | 0.731 |
| **NEO tau** |  |  |  |  |  |
|  | Amyloid burden | 0.675 (0.215 to 1.134) | **0.004** | 0.464 (0.108 to 0.819) | **0.011** |
|  | Age | 0.222 (-0.024 to 0.468) | 0.077 | 0.084 (-0.107 to 0.274) | 0.389 |
|  | Sex | -1.004 (-1.438 to -0.57) | **<0.001** | -0.913 (-1.262 to -0.565) | **<0.001** |
|  | time diff. PET scans | 0.08 (-0.165 to 0.325) | 0.523 | 0.049 (-0.146 to 0.245) | 0.621 |
|  | APOE4 | 0.151 (-0.273 to 0.576) | 0.485 | 0.186 (-0.141 to 0.512) | 0.265 |
| **Amyloid burden** |  |  |  |  |  |
|  | Age | 0.098 (0.018 to 0.179) | **0.016** | 0.436 (0.21 to 0.663) | **<0.001** |
|  | APOE4 | 0.334 (0.12 to 0.548) | **0.002** | 0.427 (0.022 to 0.831) | **0.039** |
|  | Sex | 0.267 (0.109 to 0.424) | **0.001** | 0.2 (-0.146 to 0.546) | 0.257 |

**STable 8. Structural equation model parameter estimates for episodic memory sub-model including medial temporal lobe and neocortical tau burden variables.**

For the model with a continuous amyloid variable, only the estimates of the paths to "MTL tau," "NEO tau," and "Amyloid burden" are reported, because the remaining estimates are virtually identical to those reported for the model with a dichotomous amyloid variable. Parameter estimates are unstandardized so that estimates for the same path can be compared across models. “Sex” was coded as women = 0 and men = 1. Abbreviations: MEM = episodic memory; EC = entorhinal cortex; PhC = parahippocampal cortex; ICV = intracranial volume; MTL = medial temporal lobe; NEO = neocortical; CL = Centiloid.


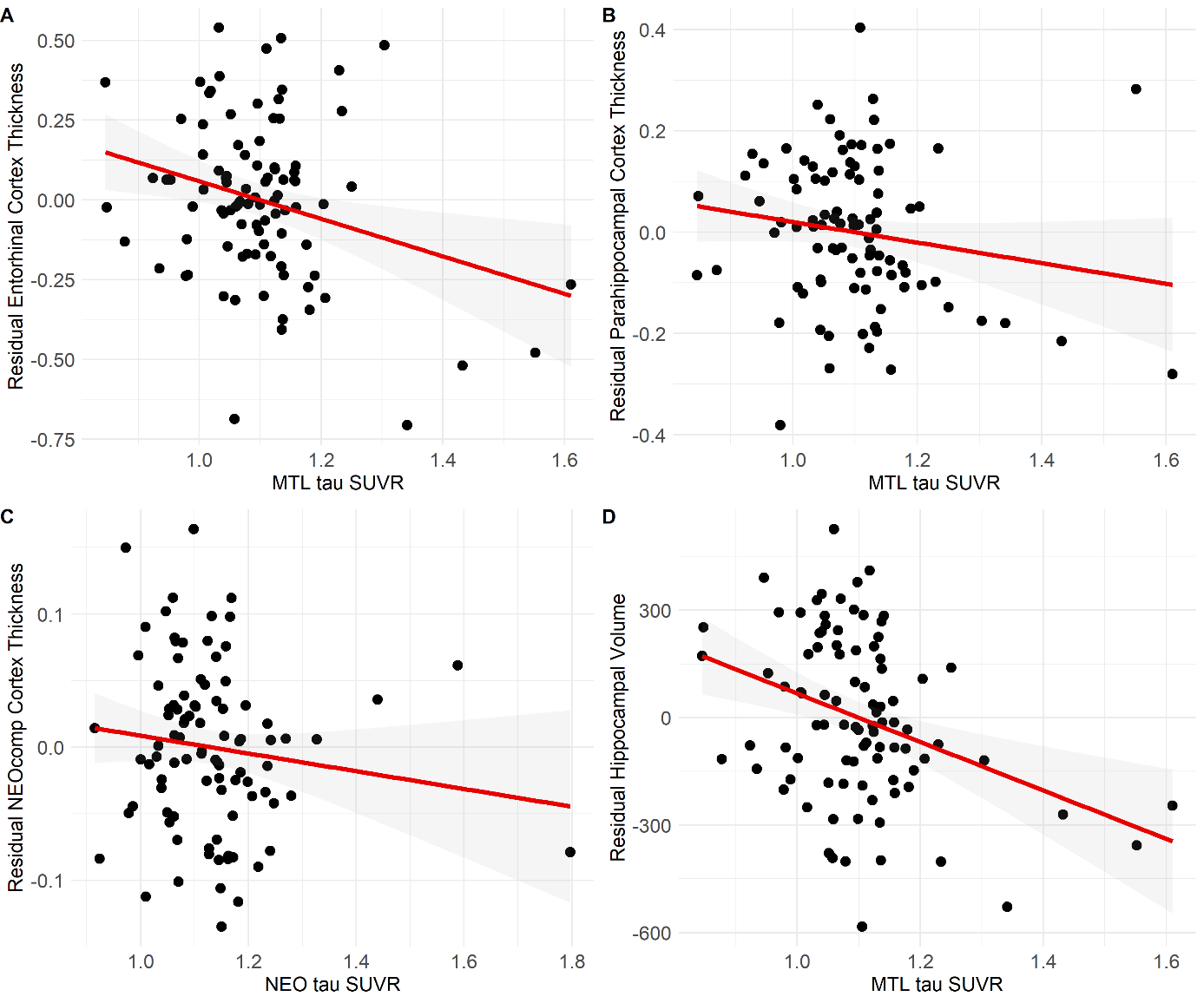


**SFigure 3. Correlation plots demonstrating associations between tau burden and cortical thickness/volume of the investigated ROIs.** The values in the graphs are the residuals of entorhinal cortex thickness (A), parahippocampal cortex thickness (B), NEOcomp cortex thickness (C), and hippocampal volume (D) after regressing out the effects of age and sex and, in the case of hippocampal volume, additionally of total intracranial volume. Abbreviations: NEO = neocortical; MTL = medial temporal lobe; SUVR = standardized uptake value ratio.


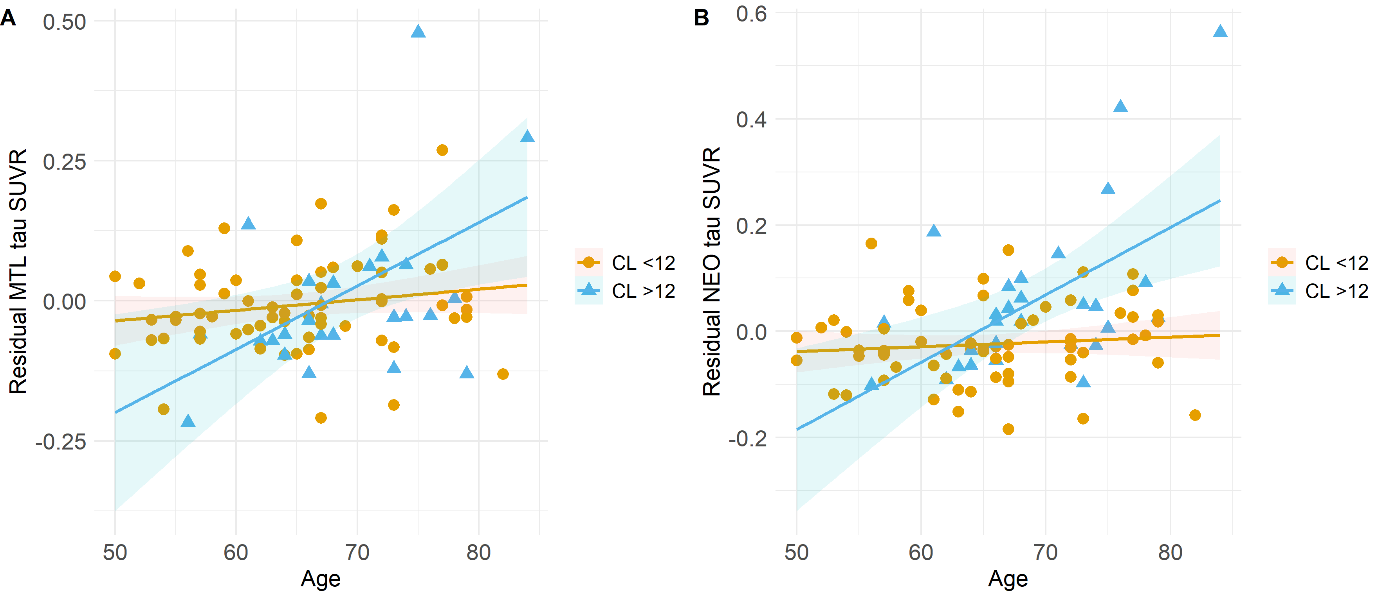


**SFigure 4. Correlation plots demonstrating the associations between age and tau burden depending on Centiloid value and brain region.** The values in the graphs are the residuals of MTL tau (A) and NEO tau (B) after regressing out the effects of age, sex, APOE4 and time between PET scans. Abbreviations: CL = Centiloid; NEO = neocortical; MTL = medial temporal lobe; SUVR = standardized uptake value ratio.

**STable 9. Structural equation model parameter estimates for episodic memory sub-model including single medial temporal lobe tau variables.**

| **Regressions** | **derived from** | **β** | **95% CI** | ***P*-value** |
| --- | --- | --- | --- | --- |
| **MEM** |  |  |  |  |
|  | EC thickness | 0.022 | -0.131 to 0.175 | 0.781 |
|  | PhC thickness | 0.094 | -0.042 to 0.230 | 0.175 |
|  | Hippocampal volume | 0.236 | 0.014 to 0.457 | **0.037** |
|  | EC tau | -0.18 | -0.36 to -0.001 | **0.049** |
|  | Age | -0.046 | -0.202 to 0.111 | 0.566 |
|  | Years of Education | 0.124 | -0.02 to 0.267 | 0.092 |
|  | Sex | -0.743 | -1.157 to -0.328 | **<0.001** |
|  | total ICV | -0.088 | -0.295 to 0.118 | 0.403 |
|  | time diff. PET scans | 0.027 | -0.295 to 0.118 | 0.67 |
| **EC thickness** |  |  |  |  |
|  | EC tau | -0.319 | -0.524 to -0.113 | **0.002** |
|  | Age | -0.077 | -0.256 to 0.102 | 0.398 |
|  | Sex | 0.267 | -0.131 to 0.664 | 0.188 |
| **PhC thickness** |  |  |  |  |
|  | PhC tau | -0.242 | -0.449 to -0.035 | **0.022** |
|  | Age | -0.071 | -0.275 to 0.113 | 0.496 |
|  | Sex | -0.016 | -0.445 to 0.413 | 0.942 |
| **Hippocampal volume** |  |  |  |  |
|  | Amygdala tau | -0.28 | -0.385 to -0.176 | **<0.001** |
|  | Age | -0.224 | -0.344 to -0.103 | **<0.001** |
|  | Sex | 0.06 | -0.274 to 0.394 | 0.726 |
|  | total ICV | 0.587 | 0.411 to 0.763 | **<0.001** |
| **EC tau** |  |  |  |  |
|  | Age | 0.183 | -0.017 to 0.384 | 0.073 |
|  | Sex | -0.727 | -1.187 to -0.267 | **0.002** |
|  | Amyloid >12CL | 0.136 | -0.406 to 0.678 | 0.624 |
| **PhC tau** |  |  |  |  |
|  | Age | 0.195 | -0.035 to 0.425 | 0.097 |
|  | Sex | -0.740 | -1.219 to -0.26 | **0.003** |
|  | Amyloid >12CL | 0.354 | -0.189 to 0.896 | 0.201 |
| **Amygdala tau** |  |  |  |  |
|  | Age | 0.289 | 0.068 to 0.51 | **0.01** |
|  | Sex | -0.44 | -0.82 to -0.059 | **0.023** |
|  | Amyloid >12CL | 0.14 | -0.363 to 0.642 | 0.586 |
| **Amyloid >12CL** |  |  |  |  |
|  | Age | 0.091 | 0.006 to 0.176 | **0.036** |
|  | Sex | 0.238 | 0.068 to 0.408 | **0.006** |

Model parameter estimates are unstandardized. “Sex” was coded as women = 0 and men = 1. Abbreviations: MEM = episodic memory; EC = entorhinal cortex; PhC = parahippocampal cortex; ICV = intracranial volume; MTL = medial temporal lobe; NEO = neocortical; CL = Centiloid.


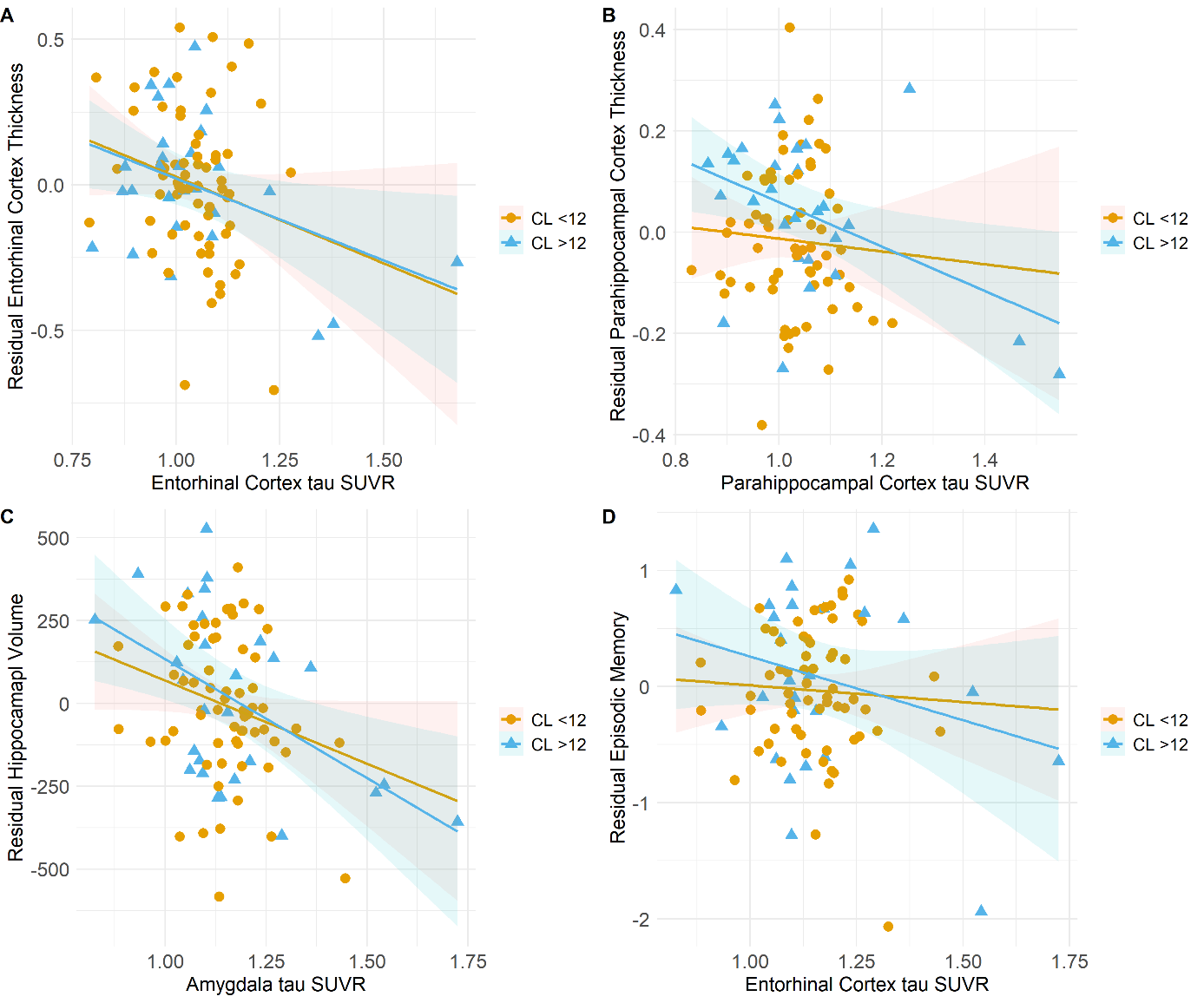


**SFigure 5. Correlation plots demonstrating the associations between reginal tau burden and regional thickness/volume (A, B, C) as well as episodic memory (D) depending on Centiloid value and brain region.** The values in the graphs are the residuals of entorhinal cortex thickness (A), parahippocampal cortex thickness (B), and hippocampal volume (D) after regressing out the effects of age and sex and, in the case of hippocampal volume, additionally of total intracranial volume. Episodic memory performance (D) is adjusted for age, sex, education, time between PET scans, and hippocampal volume. Two participants with a missing neuropsychological test included in the episodic memory composite score are not included in the plot but were included in the analysis. Abbreviations: CL = Centiloid; SUVR = standardized uptake value ratio.

**STable 10. Indices of Fit for Structural Equation Models.**

|  | | **Statistical index of fit** | | | **Practical Indices of fit** | | | |
| --- | --- | --- | --- | --- | --- | --- | --- | --- |
| **Model** | **Description** | **χ^2^** | **df** | ***P*-value** | **RMSEA [90% CI]** | **SRMR** | **CFI** | **TLI** |
| 1: STable 4 | total cohort, global cognition as outcome variable | 97.0 | 55 | <0.001 | 0.057 [0.038 to 0.076] | 0.045 | 0.96 | 0.90 |
| 2: STable 4 | CU only, global cognition as outcome variable | 107.8 | 55 | <0.001 | 0.071 [0.051 to 0.091] | 0.054 | 0.92 | 0.82 |
| 3: STable 5 | total cohort, EXE and MEM as outcome variable | 297.1 | 191 | <0.001 | 0.048 [0.037 to 0.059] | 0.048 | 0.96 | 0.94 |
| 4: STable 5 | CU only, EXE and MEM as outcome variable | 259.0 | 191 | 0.001 | 0.044 [0.029 to 0.057] | 0.051 | 0.96 | 0.94 |
| 5: STable 6 | Multigroup EXE sub-model | 138.4 | 94 | 0.002 | 0.064 [0.039 to 0.086] | 0.072 | 0.92 | 0.90 |
| 6: STable 7 | Multigroup MEM sub-model | 232.3 | 170 | 0.001 | 0.056 [0.037 to 0.073] | 0.077 | 0.97 | 0.96 |
| 7: STable 8 | MEM sub-model including composite tau ROIs, cont. Aβ | 169.4 | 119 | 0.002 | 0.064 [0.041 to 0.087] | 0.062 | 0.94 | 0.92 |
| 8: STable 8 | MEM sub-model including composite tau ROIs, dich. Aβ | 170.5 | 119 | 0.001 | 0.066 [0.042 to 0.088] | 0.064 | 0.94 | 0.91 |
| 9: STable 9 | MEM sub-model including single MTL ROIs, dich. Aβ | 177.6 | 112 | <0.001 | 0.077 [0.055 to 0.098] | 0.064 | 0.94 | 0.91 |

A good model fit is indicated by non-significant χ^2^ statistics (p > 0.05), RMSEA < 0.05, SRMR < 0.08, CFI > 0.95, and TLI > 0.95 (4). However, note that the model fit also depends on the sample size, model complexity, and number of variables (5, 6, 7).

**Investigating [18F]-flortaucipir off-target binding in skull/meninges**


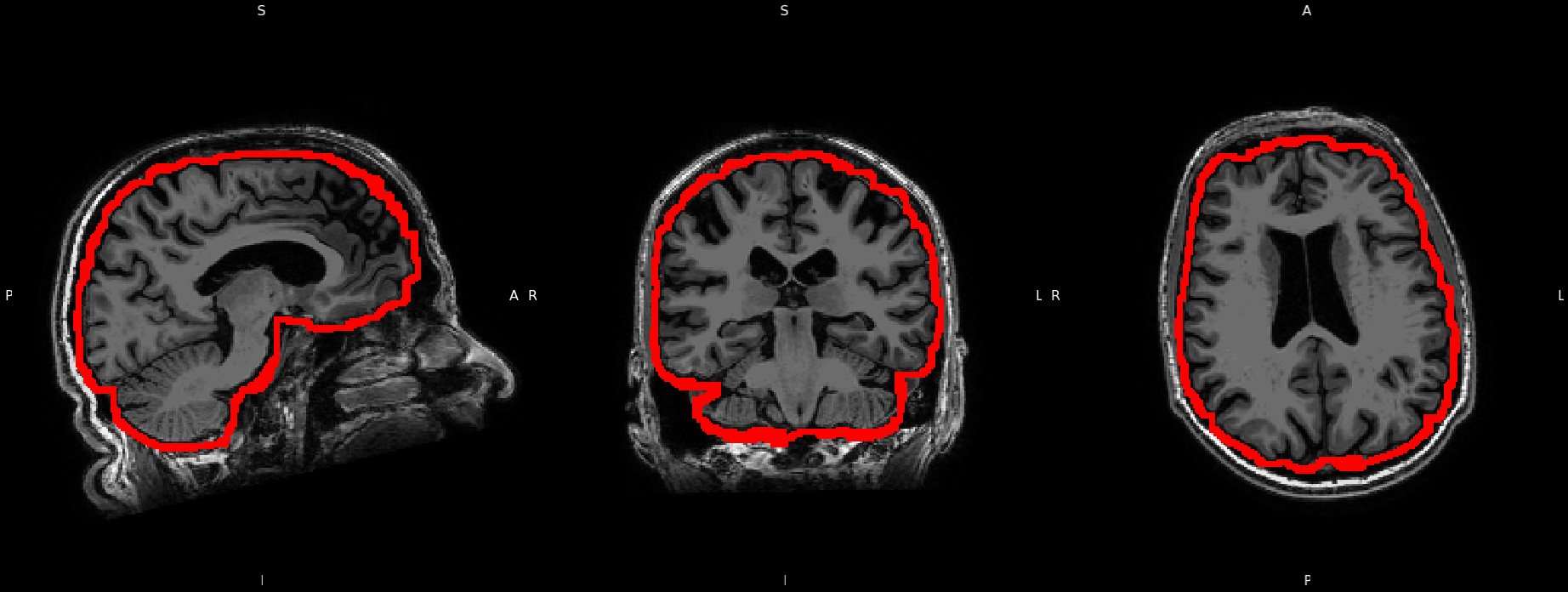
**SFigure 6.** The generated skull/meningeal ROI on a participant’s T1 image. The mask was generated following previous work by Smith and colleagues who reported sex differences in [18F]-flortaucipir binding in a skull/meningeal ROI (8).


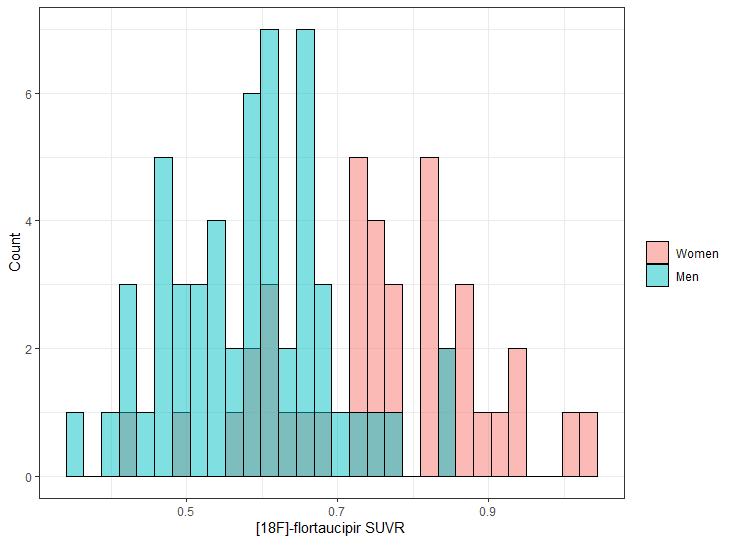


**SFigure 7.** Histogram showing the differences between male and female [18F]-flortaucipir binding in the skull/meninges mask (SUVR). Women showed significantly higher [18F]-flortaucipir binding in the skull/meninges mask (t = 6.922, df = 91, p <0.001).

**Supplementary References**

1. Hedden T, Schultz AP, Rieckmann A, Mormino EC, Johnson KA, Sperling RA, et al. Multiple Brain Markers are Linked to Age-Related Variation in Cognition. Cereb Cortex. 2016;26(4):1388-400.

2. Milfont TL, Fischer R. Testing measurement invariance across groups: applications in cross-cultural research. International Journal of Psychological Research. 2010;3(1):111-30.

3. Cheung GW, Rensvold RB. Testing factorial invariance across groups: A reconceptualization and proposed new method. Journal of management. 1999;25(1):1-27.

4. Schermelleh-Engel K, Moosbrugger H, Müller H. Evaluating the fit of structural equation models: Tests of significance and descriptive goodness-of-fit measures. Methods of psychological research online. 2003;8(2):23-74.

5. Shi D, Lee T, Maydeu-Olivares A. Understanding the Model Size Effect on SEM Fit Indices. Educational and Psychological Measurement. 2019;79(2):310-34.

6. Kenny DA, McCoach DB. Effect of the Number of Variables on Measures of Fit in Structural Equation Modeling. Structural Equation Modeling: A Multidisciplinary Journal. 2003;10(3):333-51.

7. Kenny DA, Kaniskan B, McCoach DB. The Performance of RMSEA in Models With Small Degrees of Freedom. Sociological Methods & Research. 2015;44(3):486-507.

8. Smith R, Strandberg O, Leuzy A, Betthauser TJ, Johnson SC, Pereira JB, et al. Sex differences in off-target binding using tau positron emission tomography. Neuroimage Clin. 2021;31:102708.
